# Inter-kingdom relationships in Crohn’s disease explored using a multi-omics approach

**DOI:** 10.1080/19490976.2021.1930871

**Published:** 2021-07-09

**Authors:** Alessandra Frau, Umer Z. Ijaz, Rachael Slater, Daisy Jonkers, John Penders, Barry J. Campbell, John G. Kenny, Neil Hall, Luca Lenzi, Michael D. Burkitt, Marieke Pierik, Alistair C. Darby, Christopher S. J. Probert

**Affiliations:** a Department of Molecular and Clinical Cancer Medicine, Institute of Systems, Molecular and Integrative Biology, University of Liverpool, Liverpool, UK; b School of Engineering, University of Glasgow, Glasgow, UK; c School of Nutrition and Translational Research in Metabolism, Maastricht University, Maastricht, Netherlands; d Department of Infection & Microbiomes, University of Liverpool, Liverpool, UK; e Teagasc Food Research Centre, Cork, Ireland; f Earlham Institute, Norwich, UK; g School of Biological Sciences, University of East Anglia, Norwich, Norfolk, UK; h Centre for Genomic Research, University of Liverpool, Liverpool, UK; i Division of Diabetes, Endocrinology and Gastroenterology, University of Manchester, Manchester, UK

**Keywords:** Crohn’s disease, metabolome, microbiome, mycobiome, volatile organic compound

## Abstract

The etiology of Crohn’s disease (CD) is multifactorial. Bacterial and fungal microbiota are involved in the onset and/or progression of the disease. A bacterial dysbiosis in CD patients is accepted; however, less is known about the mycobiome and the relationships between the two communities. We investigated the interkingdom relationships, their metabolic consequences, and the changes in the fungal community during relapse and remission in CD.

Two cohorts were evaluated: a British cohort (n = 63) comprising CD and ulcerative colitis patients, and controls. The fungal and bacterial communities of biopsy and fecal samples were analyzed, with the fecal volatiles; datasets were also integrated; and a Dutch cohort (n = 41) comprising CD patients and healthy controls was analyzed for stability of the gut mycobiome.

A dysbiosis of the bacterial community was observed in biopsies and stool. Results suggest *Bacteroides* is likely key in CD and may modulate *Candida* colonization. A dysbiosis of the fungal community was observed only in the Dutch cohort; *Malassezia* and *Candida* were increased in patients taking immunosuppressants. Longitudinal analysis showed an increase in *Cyberlindnera* in relapse. *Saccharomyces* was dominant in all fecal samples, but not in biopsies, some of which did not yield fungal reads; amino acid degradation was the main metabolic change associated with CD and both bacteria and fungi might be implicated.

We have shown that *Bacteroides* and yeasts may play a role in CD; understanding their role and relationship in the disease would shed new light on the development and treatment of CD.

## Introduction

The etiology of inflammatory bowel disease (IBD) is multifactorial. There are perturbations of immune regulation and the microbiota,^1^ and over 200 risk alleles.^2^ The incidence of IBD has increased with Westernization: in countries where IBD was rare, the incidence is approaching that in Europe.^3^ Environmental factors influence the intestinal microbiota.^4^ Many observations suggest a link between fungi and IBD. There are increased fungal metabolites in the feces of patients with active Crohn’s disease (CD).^5^ Anti-*Saccharomyces cerevisiae* antibodies (ASCA) in CD patients are linked with genetic mutations involved in the immune response to fungi and IBD.^6^ Many studies have reported changes in the bacterial community in IBD^7,8^ but fewer have looked at the role of fungi in IBD.^9–19^ In general, the mycobiome of IBD patients contains significantly more *Candida* than that of healthy controls^11–14^ and an increase of *Malassezia* has been reported in CD patients.^18^ A recent study has also shown that *Debaryomyces* (a yeast commonly found in food products) prevents healing in the inflamed mucosa of CD patients.^20^

Murine studies support a role for fungi in IBD. Iliev *et al*.^21^ demonstrated that ASCA increases in a murine colitis model induced by ingestion of dextran sodium sulfate (DSS). Analysis of the fecal mycobiome showed that *C. tropicalis* dominated the community in DSS-treated mice, and nonpathogenic *Saccharomyces* was decreased. Tissue damage appears to be regulated by Dectin-1, a C-type lectin receptor used for fungal cell wall recognition. Increased susceptibility to DSS and increased fungal burden (*Candida*) was also observed in Dectin-3-deficient mice.^22^ Susceptibility to colitis in Dectin-1 deficient mice was ameliorated by the antifungal agent fluconazole. This is controversial because broad-spectrum antifungals may exacerbate colitis^23,24^ and increase bacterial diversity, resulting in increases of pathogenic species.^23^ A study on CX3CR1+ expressing mononuclear phagocytes (MNPs) reported that these are involved in the immune response to intestinal fungi; selective depletion of intestinal CX3CR1+ MNPs in mice treated with DSS was associated with severe colitis (ameliorated by the use of fluconazole); and an impaired immune response to fungi in CD subjects with a polymorphism of CX3CR1 gene.^25^ These studies imply that fungi have a complex role in IBD. They also indicate an interaction between fungi and bacteria. Interkingdom communications in the intestinal environment, where organisms are physically closely located, appears intuitive. Indeed, both positive^13,26^ and negative^27^ connections between bacteria and fungi have been reported (summarized in^28^).

Microbiome studies are typically costly and incur confounding technical challenges. The fecal volatile metabolome is relatively inexpensive to investigate. Biomarker amplicon sequencing focuses on the composition of a community. The metabolome can potentially provide a clearer indication of the functional activity of a microbial community. We have undertaken metabolomics studies of volatile compounds from feces of patients with IBD and other disorders,^29,30^ and reported the changes in volatile organic compounds (VOCs) associated with CD and ulcerative colitis (UC).^5^ Some of these metabolites appeared to be of fungal origin^5^ and this led us to investigate the fecal mycobiome of patients with IBD.

The need to integrate multiple high-dimensional biology approaches to further understanding of the function of gut microbiota in IBD has been proposed.^1^ A recent study showed the potential of combining omics approach to integrate metabolomics and microbiome results.^31^ Other studies have investigated the bacterial and fungal community of the same cohort (e.g.^12,13^), and recently a study combined metabolomics data to bacterial and fungal community datasets.^19^ In here, a similar multi-omics approach was undertaken to explore the underlying biological mechanisms, using an optimized genomic analysis of the fecal mycobiome of patients with IBD^32^ and a dedicated tool to integrate datasets.^33^

This study investigated the hypothesis that interactions between fungal and bacterial communities and consequential metabolic changes would be observed in patients with CD, and that the mycobiome would evolve during periods of disease activity and remission. Specifically, we performed a cross-sectional analysis of a British cohort of 23 patients with CD, 20 with UC and 20 non-IBD controls. In addition, we analyzed the fungal community longitudinally, in a Dutch cohort of CD patients (n = 26), sampled multiple times in relapse and remission, and healthy controls (n = 15). Analysis of the bacterial community of the Dutch cohort was beyond the scope of this work and it has been already published in a separate study.^34^ We investigated the fecal microbiome samples from both cohorts of IBD patients and the mucosal microbiome and fecal volatile metabolome from the British cohort. State-of-the-art multi-omics integration tools were deployed.

## Results

### Patients’ demographics

The demographic and clinical characteristics of the British cohort are given in Table 1. Stool samples from 63 subjects were analyzed. For a subset of patients (n = 41), biopsies from the ileum (n = 34) and/or the colon (n = 80) were analyzed. Active disease was defined based on fecal calprotectin (>200 μg/g) for both CD and UC.

**Table 1.** Demographic of the participants from the British cohort. Continuous variables are expressed as median (interquartile range, IQR), the other variables (sex, smoking, distribution and medications use) are shown as n and percentage. L1 = ileal CD, L2 = colonic CD, L3 = ileo-colonic distribution of CD. BMI = body mass index, TI = terminal ileum, TC = transverse colon, SC = sigmoid colon, bdl = below detection limit. * refers to the whole cohort of 63 subjects. ** use of antibacterial drugs in the 3 months prior collection of the sample

	CROHN’S DISEASE		ULCERATIVE COLITIS		CONTROLS (NON-IBD)
Activity Status	Remission	Active	Remission	Active	
**Subjects – Stool**	**n = 12**	**n = 11**	**n = 11**	**n = 9**	**n = 20**
Sex	F = 9 (75%)	F = 5 (45.4%)	F = 5 (45.4%)	F = 3 (33.3%)	F = 9 (45%)
Age (median, IQR)	33.5 (17)	32 (31)	47 (29)	50 (12)	55 (21)
BMI (median, IQR)	25 (6)	22 (5)	27 (4)	26.215 (6.5)	28.28 (6)
Smoking (Yes or previous)	8 (66.7%)	4 (36.4%)	4 (36.4%)	4 (44.4%)	8 (40%)
Fecal calprotectin (μg/g) (median)	84	376	49	368	42
**Subjects – Biopsies**	**n = 8**	**n = 10**	**n = 8**	**n = 4**	**n = 11**
Sex	F = 5 (62.5%)	F = 5 (50%)	F = 3 (37.5%)	F = 2 (50%)	F = 7 (63.6%)
Age (median, IQR)	33.5 (14.5)	36.5 (29)	45.5 (18.5)	52.5 (16)	56 (26)
BMI (median, IQR)	25 (3)	22 (5)	27.955 (3)	24.43 (6)	26.33 (5)
Smoking status (Yes/previous)	3 (37.5%)	3 (30%)	3 (37.5%)	1 (25%)	5 (45.4%)
Disease distribution	L2 = 4 (50%) L3 = 4 (50%)	L1 = 4 (40%) L3 = 6 (60%)	-	-	-
Biopsy site	TI = 8 TC = 8 SC = 7	TI = 7 TC = 10 SC = 10	TI = 8 TC = 8 SC = 8	TI = 3 TC = 4 SC = 4	TI = 8 TC = 11 SC = 10
**Subjects – Plasma**	**n = 8**	**n = 8**	**n = 10**	**n = 8**	**n = 14**
ASCA (RU/ml)	46.995 (97.38)	24.485 (113.77)	Bdl (bdl-6.23)	2.835 (bdl-8.61)	5.72 (bdl-17.75)
Medications*					
Antibiotics**	n = 1 (8.33%)	n = 2 (18.18%)	n = 0	n = 0	n = 1 (5%)
Mesalamine	n = 2 (16.67%)	n = 2 (18.18%)	n = 9 (81.81%)	n = 6 (66.67%)	n = 0
Glucocorticoids	n = 1 (8.33%)	n = 2 (18.18%)	n = 0	n = 1 (11.11%)	n = 2 (10%)
Immunosuppressants	n = 2 (16.67%)	n = 4 (36.36%)	n = 1 (9.09%)	n = 3 (33.33%)	n = 1 (5%)
Biologics	n = 0	n = 3 (27.27%)	n = 2 (18.18%)	n = 2 (22.22%)	n = 0
Selective Serotinin Reuptake Inhibitor	n = 1 (8.33%)	n = 1 (9.09%)	n = 1 (9.09%)	n = 0	n = 3 (15%)
Proton pump inhibitor	n = 2 (16.67%)	n = 3 (27.27%)	n = 2 (18.18%)	n = 0	n = 5 (25%)

**Table 2.** Demographic of the participants from the Dutch cohort at baseline (n = 41). Continuous variables are expressed as median (interquartile range, IQR), the other variables (sex, smoking, status and distribution) are shown as n and percentage. L1 = ileal CD, L2 = colonic CD, L3 = ileo-colonic distribution of CD

	CROHN’S DISEASE	HEALTHY CONTROLS
Subjects	n = 26	n = 15
Sex	F = 14 (53.8%)	F = 7 (46.7%)
Age (median, IQR)	43.5 (34)	25 (7)
Smoking status (Yes/previous)	14 (53.8%)	2 (13.3%)
Status	Active = 8 (30.8%) Remission = 18 (69.2%)	-
Distribution	L1 = 9 (34.6%) L2 = 7 (36.9%) L3 = 9 (34.6%)	-

**Table 3.** Medication use and clinical data (Dutch cohort) for remission and active samples (n = 44). T1 = time point 1, T2 = time point 2. Continuous variables are expressed as median (interquartile range, IQR), the other variables (distribution and medications use) are shown as n and percentage. L1 = ileal, L2 = colonic, L3 = ileo-colonic distribution of CD. CRP = C-reactive protein. * use of antibiotics in the 2 months prior collection of the sample

	T1 Remission (n = 22)	T2 Remission (n = 12)	T2 Active (n = 10)
Distribution	L1 = 10 (45.4%) L2 = 5 (22.7%) L3 = 7 (31.8%)	L1 = 5 (41.67%) L2 = 2 (16.67%) L3 = 5 (41.67%)	L1 = 5 (50%) L2 = 3 (30%) L3 = 2 (20%)
Fecal calprotectin (μg/g) (median, IQR)	43 (45.5)	38.5 (62.5)	252 (80)
Serum CRP	1.9 (1.75)	1.5 (1.3)	3.5 (2.8)
**Medications**			
Mesalamine	n = 4 (18.2%)	n = 1 (8.33%)	n = 1 (10%)
Immunosuppressants	n = 9 (40.9%)	n = 5 (41.67%)	n = 5 (50%)
Biologics	n = 13 (59.1%)	n = 5 (41.67%)	n = 8 (80%)
Proton pump inhibitor	n = 8 (36.4%)	n = 4 (33.33%)	n = 4 (40%)
Antibiotics*	n = 1 (4.5%)	n = 1 (8.33%)	n = 0

For the Dutch cohort, 53 samples were collected from 26 CD patients during relapse and remission. Activity of CD was based on fecal calprotectin as described in Tedjo *et al*.^34^ Stool was also donated by 15 healthy individuals, 14 of these were sampled twice on different occasions. Demographic and clinical characteristics for this cohort are presented in Table 2 and 3.

### Bacterial microbiome of the British cohort

**Figure 1.**
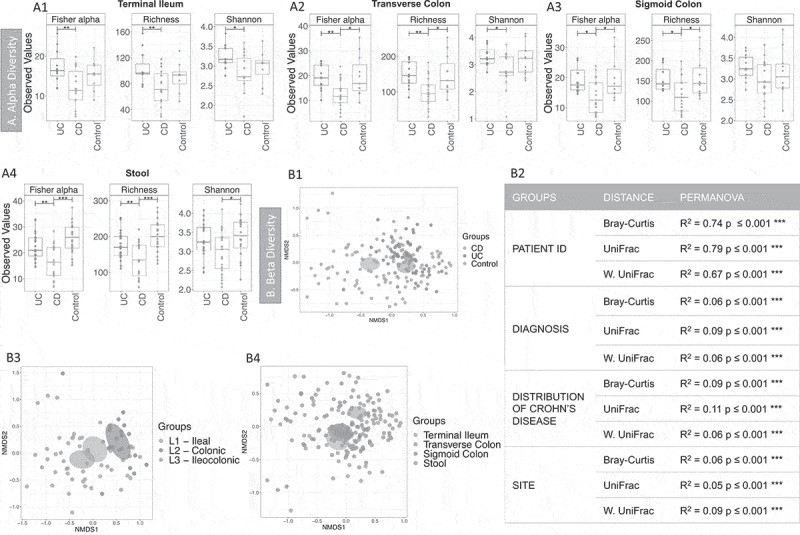
Alpha and Beta diversity results of the bacterial gut microbiome (British cohort). A1 to A4: Alpha diversity of the bacterial microbiome from the four specimen types: terminal ileum, transverse colon, sigmoid colon and stool. Samples were grouped according to diagnosis: Crohn’s disease (CD), ulcerative colitis (UC) and Controls. Three indices were considered: Fisher alpha, richness and Shannon index. Pair-wise ANOVA was calculated between the groups (CD, UC and Controls) and if significant, stars are shown on top (* *p* < .05, ** *p* < .01 and *** *p* < .001). B1 to B4: Nonmetric distance scaling (NMDS) showing clustering of samples. To produce these charts, samples (n = 176) from patients for which terminal ileum (TI), transverse colon (TC), sigmoid colon (SC) and stool were available. In B3 only samples from CD patients (n = 72) were considered. The charts were produced using Bray–Curtis (Operational taxonomy units (OTUs) level). The ellipses represent 95% confidence interval of standard error for a given group. The table in B2 summarizes permutational multivariate analysis of variance (PERMANOVA) results for all the distances, Bray–Curtis, unweighted UniFrac (UniFrac) and weighted (W. UniFrac). R^2^ refers to the percentage of variability among samples’ microbiome that can be explained by that factor/metadata

The bacterial community of stool and biopsy samples was analyzed. After filtering, the median number of reads per sample was 100,905.5 (min 4401, max 2,511,360 reads). Alpha diversity, which looks at and compares groups in terms of number for species and their abundance, (Figure 1-A) was lower in CD (biopsy and stool) compared with control and/or UC, but this was not observed when comparing controls and UC patients.

Sample similarity was explored using beta diversity, that is, how similar samples were in terms of distribution of taxa (using Bray-curtis distance), or phylogeny (unweighted Unifrac distance), or a combination of both (weighted Unifrac distance) (Figure 1-B). Permutational multivariate analysis of variance (PERMANOVA) analysis (Figure 1-B2) showed that the main sources of variability in community structure were the patient ID, followed by the diagnosis and the site/specimen type. Samples clustered according to the diagnosis (Figure 1-B1) and the distribution of the disease (Figure 1-B3), with clear segregation between patients with exclusively ileal or colonic disease. PERMANOVA analysis was also performed for all the predictors (Supplemental material 2, Table S1). Significant variability in community structure was found for age, BMI, sex, ASCA, antibiotics, mesalamine- and immunosuppressant-use. Significant results were obtained for the use of antibiotics, but no sample was excluded, as none was an outlier (see Supplemental material 3, Figure S1).

**Figure 2.**
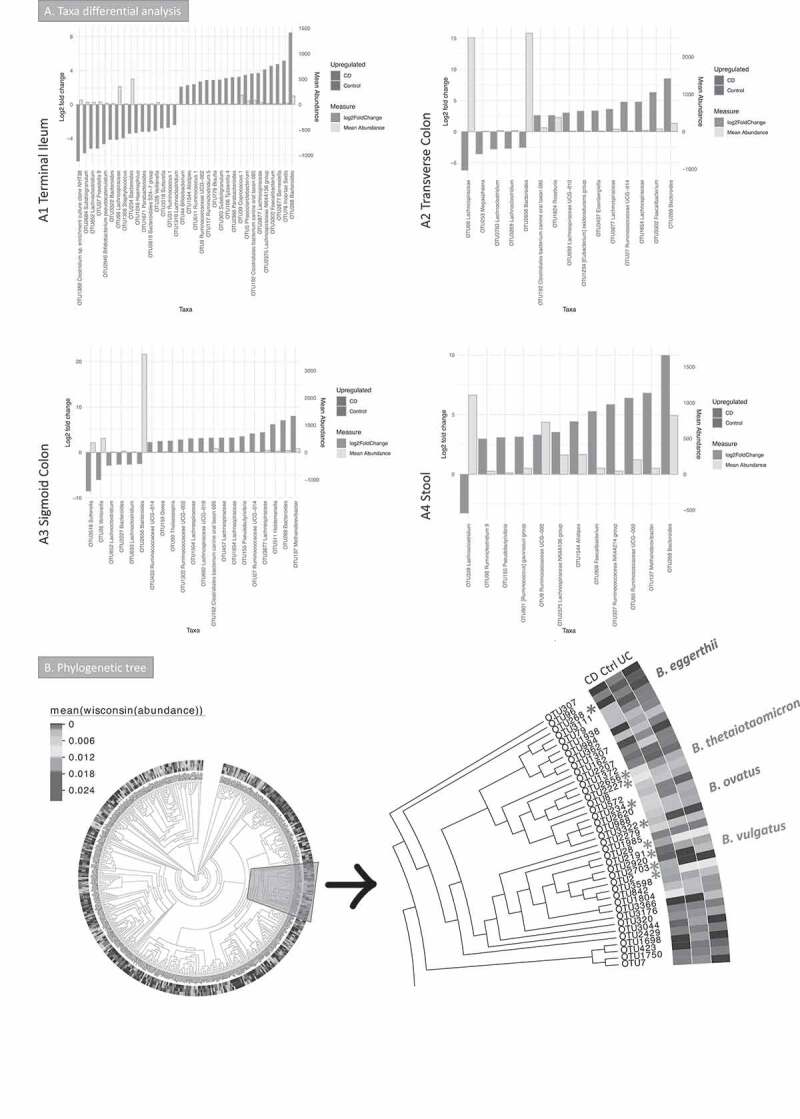
A Taxa differential analysis results and phylogenetic tree of the bacterial gut microbiome (British cohort). A Taxa differential analysis comparing CD and control samples. Results are presented according to sample type: A1: Terminal Ileum (TI); A2: Transverse colon (TC); A3: sigmoid colon (SC); A4: stool. The bar charts show Log2 fold change in abundance between groups (y axis on the left and dark gray bar) and the mean abundance across all the samples (y axis on the right and light gray bar). Taxa increased in CD patients have bars with a red border, meanwhile taxa increased in controls have bars with a blue border. A subset of the most important and abundant OTUs selected using Random Forest are shown; these are taxa that allowed to achieve a 70–80% of accuracy in discriminating between groups are shown (details are in Supplemental material 1). A complete list of taxa that had significant different abundance, and further details, including adjusted *p* values, is in Supplemental material 4. B Phylogenetic tree of bacterial 16S rRNA OTUs. Samples (n = 176) were from TI, TC, SC and stool (British cohort) with the *Bacteroides* branch magnified. The tree was visualized with EvolView, which was also used to add the heatmap. The * indicates OTUs that were increased in the groups analyzed or selected in the subsets (BV-STEP analysis) that best correlated with the whole OTU table

Taxa differential analysis (enrichment analysis to highlight taxa that are log2 fold different) results are shown in Figure 2. Operational taxonomy units (OTUs) assigned to *Lachnoclostridium* were increased in all mucosa sites and the stool in CD. Some OTUs assigned to *Bacteroides* were increased in the mucosa in CD, whereas others were decreased. *Bacteroides* was decreased in stool samples in CD. The butyrate producers *Faecalibacterium* and *Roseburia* were depleted in CD in most of the sites analyzed. The same was observed for *Methanobrevibacter*.

Taxonomy summary and environmental filtering results are shown in Supplemental material 3, Figure S2. Environmental filtering assumes that microbial communities under a stronger environmental pressure are more phylogenetically similar (clustered) either on a global scale (net relatedness index, NRI) or in terms of terminal clades (nearest taxon index, NTI), as closer taxa phylogenies are likely to share similar functionality. Interestingly, CD patients had higher values of both phylogenetic alpha diversity measures NRI and NTI than controls (stool and colon), and UC (all sites) implying that CD creates an environmental pressure on the microbiota (Supplemental material 3, Figure S2).

BV-STEP routine, as used previously by the authors,^35–37^ was applied to reduce the complexity of the OTU table and find subsets of relevant OTUs. This algorithm calculates distances (Bray–Curtis) between samples by permuting through the subsets of OTUs in such a way that the distances (using fewer OTUs) remain roughly conserved in beta diversity space with the distances between samples using all the OTUs, that is, the correlation is maximized between the distances obtained from reduced subset of OTUs with that of full set of OTUs. PERMANOVA was used on the subsets obtained to see if these have discriminatory power, and whether calprotectin (an indicator of intestinal inflammation) could explain the variability of microbial communities based on these subsets (Supplemental material 2, Table S2). Seven subsets were found with the OTU table from stool, with a correlation (R) > 0.6. Up to 16% of the variability of five of these subsets could be explained by calprotectin. Similar subsets were obtained for the terminal ileum, transverse and sigmoid colon; *Bacteroides* OTUs were often found in the mucosa subsets, along with butyrate producers (*Dorea, Roseburia, Faecalibacterium* and *Blautia*) and *Escherichia-Shigella* (see Supplemental material 2, Table S2).

Taxa differential analysis and BV-STEP showed that some *Bacteroides* OTUs were more relevant in CD, whilst others were more abundant in controls. In order to understand whether the OTUs increased in one group (CD or control) or site (mucosa sites or stool) where different from those increased in the others, further analysis was carried out. The Evolview website was used to visualize the phylogenetic tree with the *Bacteroides* branch (Figure 2-C) isolated and magnified. These results suggested that these OTUs were phylogenetically different. These OTUs were subsequently blasted against NCBI NT database (see methods). OTUs that were significantly increased in CD cohort were assigned to *B. ovatus* (OTU234 and OTU3322) (terminal ileum) and *B. thetaiotaomicron* (OTU2656 and OTU2227) (transverse colon). *B. eggerthii* (OTU268) was significantly more abundant in controls (TI, TC and stool). Other relevant OTUs (OTU2191, OTU2703, OTU2 and OTU28) were assigned to *B. vulgatus*. These were increased both in CD and controls compared to UC.

### Fungal microbiome of the British cohort

The majority of the 63 stool samples provided fungal reads, suggesting a strong fungal 18S rRNA signal. After filtering, the median number of reads per sample was 143,310 (min 3,480, max 975,102 reads). All biopsy samples (n = 119) were sequenced for fungal 18S rRNA; 38 yielded a low number of, or mainly spurious, reads and were discarded during analysis (see Methods and Supplemental material 3, Figure S3), leaving 81 samples. After filtering, the median number of reads per sample was 54,614 (min 1,314, max 225,544 reads).

**Figure 3.**
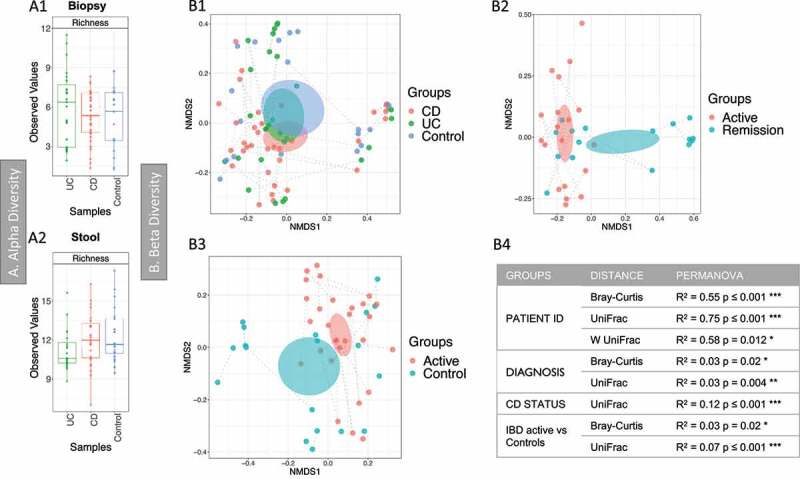
Fungal microbiome results (British cohort). Samples were from stool (n = 63) and biopsies (n = 81): terminal ileum, transverse colon and sigmoid colon. **A** Alpha diversity (richness) of samples from the mucosa (A1) and stool (A2). Samples were grouped according to diagnosis: Crohn’s disease (CD), ulcerative colitis (UC) and Controls. Pair-wise ANOVA was calculated between the groups (CD, UC and Controls) but was not significant. **B** Beta diversity analysis of the fungal microbiome from the biopsy samples. B1 to B3: Nonmetric distance scaling (NMDS) showing clustering of samples. B1 compares samples according to diagnosis. In B2 only samples from CD patients were considered, meanwhile inflammatory bowel disease (IBD) active vs Control samples are compared in B3. A dashed line links samples from the same patient. The charts were produced using unweighted UniFrac (Operational taxonomy units (OTUs) level). The ellipses represent 95% confidence interval of standard error for a given group. The table in B4 summarizes permutational multivariate analysis of variance (PERMANOVA) results for distances for which significant results were obtained. R^2^ refers to the percentage of variability among samples’ microbiome that can be explained by that factor/metadata. Only significant comparisons are presented (* *p* < .05, ** *p* < .01 and *** *p* < .001)

Alpha diversity results describing microbial richness and diversity (Figure 3-A1 and A2) found few (<20) OTUs per sample, with no significant differences between the three groups (controls, CD and UC) for Richness and the two indices (Shannon and Fisher Alpha). Beta diversity results revealed no inherent clustering based on stool samples. Analysis of all biopsy samples, using PERMANOVA analysis with unweighted UniFrac distance, found that the patient ID, diagnosis and CD status (relapse vs remission) explained the variability of the microbial community (Figure 3-B1, B2 and table in B4). A difference in community signature was observed between IBD (CD and UC) in relapse and controls (Figure 3-B3). No significant difference was observed in alpha diversity and minimal change in taxa differential analysis between these groups.

PERMANOVA analysis (Supplemental material 2, Table S3) was also performed for clinical/demographic metadata as previously described. Samples from all the sites were analyzed together (separately no significant results were obtained for most comparison), many parameters gave significant results (i.e. age, sex, smoking, calprotectin and medications); for most of these the R^2^ was low (up to 3%), non-metric multidimensional scaling (NMDS) charts were produced for all metadata that gave significant results (Supplemental material 3, Figure S4). These did not show any clustering. BV-STEP analysis was performed, but no significant results were obtained.

**Figure 4.**
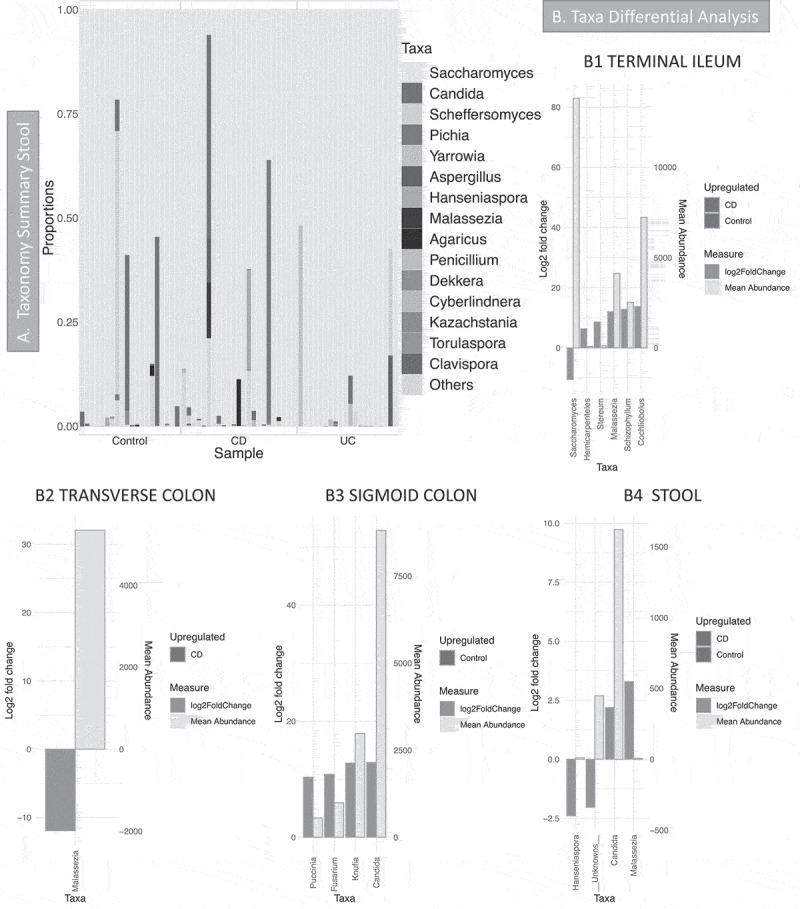
Fungal microbiome results (British cohort). Samples were from stool (n = 63) and biopsies (n = 81): terminal ileum, transverse colon and sigmoid colon. **A** Taxonomy summary for stool at genus level. **B** Taxa differential analysis (CD vs Controls) at genus level. Results are presented according to sample type: B1: Terminal Ileum samples; B2: Transverse colon samples; B3: sigmoid colon samples; B4: stool. The bar charts show Log2 fold change in abundance between groups (y axis on the left and dark gray bar) and the mean abundance across all the samples (y axis on the right and light gray bar). Taxa increased in CD patients have bars with a red border, meanwhile taxa increased in controls have bars with a blue border. Detail of these results, including adjusted *p* values, is in Supplemental material 5

Taxonomy summary of dominant taxa in stool samples (Figure 4-A) showed that *Saccharomyces* was the most common genus: it was present in every stool sample and was the most abundant genus in biopsies, but it is not present in every mucosa sample (Supplemental material 3, Figure S5). Other common genera were *Candida* and *Malassezia*. Taxa differential analysis (Figure 4-B1 to B4, details in Supplemental material 5) show that *Candida* was reduced in CD in the sigmoid colon and stool compared with controls; *Malassezia* increased in the transverse colon and stool of CD patients but was reduced in the ileum in the same patients. However, *Malassezia* abundance in stool was rather low (Figures 4B-4), and as the bar chart in Figure 4a and Supplemental material 8 show, it was observed only in a few samples.

### Fungal microbiome of the Dutch cohort

**Figure 5.**
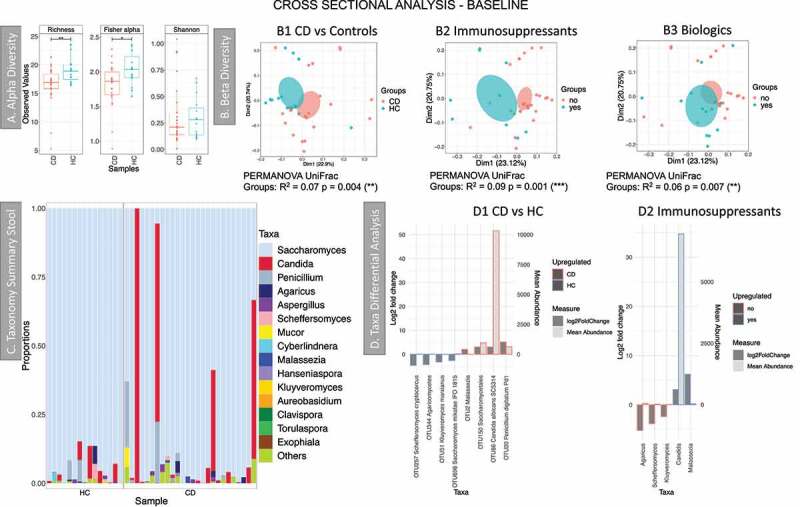
Results of the fungal microbiome analysis of the Dutch cohort comparing baseline samples (time point 1). Samples were grouped according to diagnosis: Crohn’s disease (CD, n = 26) and healthy controls (HC, n = 15). **A** Alpha diversity results (Operational taxonomy units (OTUs) level), three indices were considered: richness, Fisher alpha and Shannon. **B** Beta diversity results (OTUs level), clustering was made with Principal Coordinate Analysis (PCoA) using unweighted UniFrac distance. B1 samples grouped according to diagnosis. B2 yes = individuals given immunosuppressants (n = 11), no = individuals not taking the medication (n = 29). B3 yes = individuals given biologics (n = 12), no = individuals not taking the medication (n = 28). Permutational multivariate analysis of variance (PERMANOVA) results (R^2^ and *p* value) are reported in the bottom of each chart. **C** Taxonomy summary at genus level. **D** Taxa differential analysis, bar charts showing the OTUs that were significantly different between the two groups (HC and CD) and CD patients on Immunosuppressant (yes) vs CD patients that were not given the medication (no). The bar charts show Log2 fold change in abundance between groups (y axis on the left and dark gray bar) and the mean abundance across all the samples (y axis on the right and light gray bar). Detail of these results, including adjusted *p* values, is in Supplemental material 6

**Figure 6.**
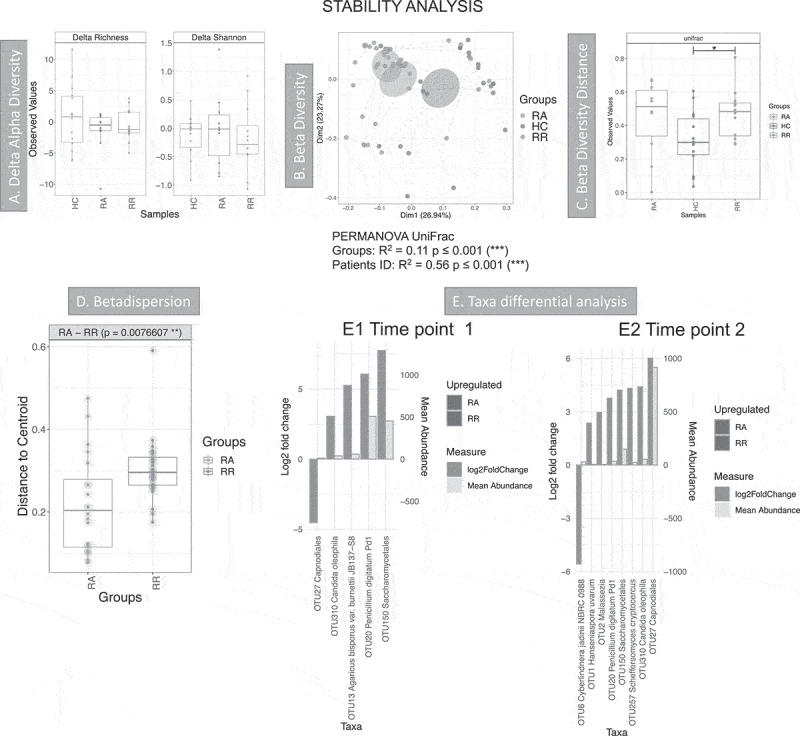
Results of the longitudinal analysis of the Dutch cohort; HC: T1 vs T2. RA: from remission to active, RR: stayed in remission. **A** Delta alpha diversity, average of changes in alpha diversity for each sample in each group. **B** Beta diversity (Principal Coordinate Analysis) describes the differences among samples/groups considering the species observed and their phylogeny (unweighted UniFrac distance). The link between points indicates that the two samples are from the same subject. The ellipses represent 95% confidence interval of standard error for a given group. PERMANOVA results (R^2^ and *p* value) are reported in the bottom of the PCoa chart. **C** Beta diversity distance describes the stability of the community during time within the same subject. A higher value indicates that there is less stability during time. Significant results are presented with an * (* *p* < .05, ** *p* < .01 and *** *p* < .001). **D** Beta-dispersion box plot comparing variance of samples from the group centroid, RA vs RR. **E** Taxa differential analysis, showing OTUs that were significantly different between RA and RR at time point 1 (E1) and RA and RR at time point 2 (E2). The bar charts show Log2 fold change in abundance between groups (y axis on the left and dark gray bar) and the mean abundance across all the samples (y axis on the right and light gray bar). Detail of these results, including adjusted *p* values, is in Supplemental material 6

The median number of reads per sample, after filtering, was 109,459.5 (min 5822, max 392,780 reads). Initial analysis compared baseline samples from healthy controls and CD patients (cross-sectional analysis) (Figure 5). Subsequently, longitudinal analysis enabled comparison of relapse and remission in the same patients (Figure 6). The comparison of healthy controls and CD patients at baseline showed that there was a dysbiosis in the fungal community in CD. This was characterized by a reduction in diversity (Figure 5-A); beta diversity analysis also showed a separation of samples between CD and controls (Figure 5-B1). The number of OTUs in these samples was low, similar to the British cohort. *Saccharomyces* was again the most abundant genus (Figure 5C) followed by *Candida*. PERMANOVA analysis, considering demographic and clinical data, gave significant results (Supplemental material 2, Table S4) for two medications: biologics and immunosuppressants. Clustering analysis (Principal Coordinate Analysis (PCoA)) showed that the samples of patients receiving these treatments formed a separate cluster (Figure 5-B2 and B3). Taxa differential analyses showed that *Saccharomyces* was significantly higher in healthy controls and *Candida* in CD patients, *Malassezia* was also increased in subjects with CD (Figure 5-D1, details in Supplemental material 6). Patients who were taking immunosuppressants had an increase of *Candida* and *Malassezia* (Figure 5-D2). As observed in the British cohort, *Malassezia* abundance was low, and it was prevalent only in a few samples (Figure 5c, Supplemental material 8).

Stability analysis compared two time points for three different groups: 1) RA patients that were in remission at time point 1 and in relapse at time point 2, 2) RR patients that were in remission at both time points and 3) HC healthy controls at two time points (T1 and T2) (Figure 6). Delta alpha diversity (Figure 6-A) calculated the changes in alpha diversity (delta) for each individual and the average changes were then compared between groups. This analysis shows that these estimates were stable over time for all groups. Overall, the community was mildly conserved within the same individual, as the ID explains almost 60% of the variability among samples (PERMANOVA results in Figure 6-B). Beta diversity estimates, describing the stability of the community during time within the same subject (Figure 6-C), also showed that the community of healthy individuals was more stable over time, and this is significant when comparing HC and RR. Beta diversity analysis (Figure 6-B), show that RA samples cluster tightly compared to RR and HC, suggesting that RA samples are similar to one another. This was also confirmed by the beta-dispersion analysis of the spread of samples (Figure 6-D), which showed that RA samples have a significantly smaller variance when compared to RR.

Taxa differential analysis showed that at T1 a *Capnodiales* OTU was more abundant in RA, but it was more abundant in RR at T2 (Figure 6-E1 and E2). *Cyberlindnera* was increased during relapse at T2 (Figure 6-E2). This suggests that *Capnodiales* may be associated with remission and that *Cyberlindnera* may thrive during inflammation.

### Metabolomic profiles of the British cohort

The metabolomics profiles of CD patients and healthy controls, from the British cohort, were compared using PLS-DA (partial least-squares projection to latent structures-discriminant analysis): CD patients formed a separate cluster (Supplemental material 3, Figure S6-A). Part of this separation was driven by two compounds related to fungal metabolism: 3,7-dimethylocta-1,6-dien-3-ol and octanal;^5,38–40^ although these may also be derived from bacteria. Branched chain fatty acids (BCFA), that is, 3-methylbutanoic acid, 2-methylpropanoic acid and 2-methylbutanoic acid, were also increased in CD, along with esters (propyl propanoate, 2-methylpropyl propanoate and 2-methylpropyl butanoate), butanoic acid, nonanal and indole (Supplemental material 3, Figure S6-B).

### Multi-omics analysis of the British cohort data

VOCs, bacterial and fungi microbiome data from stool were integrated with the DIABLO algorithm, a multi-group derivative of sparse PLS-DA. The DIABLO algorithm reduces each multivariate dataset of multiple features (VOCs, bacteria, and fungi) to components (derived by a linear combination of features in these multi-omics dataset) such that the first few components can be used to explain the majority of variability of samples. Furthermore, the algorithm has a Mfold cross-validation step to mask out outliers and gives control on the tuning of the number of components to achieve the best performance. The CD patients and controls were all compared. A model with two components was chosen: this had a classification error rate >41% (Mahalanobis distance, Supplemental material 3, Figure S7). Because of the high error rate these results were not presented. The second analysis compared CD patients in relapse and controls. The BER (balanced error rate) was lower than that in the previous comparison (around 34%, Supplemental material 3, Figure S8). Bacterial and metabolomics data were the best to discriminate between the two groups (Supplemental material 3, Figure S9-A) and showed the highest correlation for both components 1 and 2 (Supplemental material 2, Figure S9-B1 and B2). In CD in relapse, the Circo plot correlations (Supplemental material 3, Figure S9-C) showed positive correlation between BCFA (2-methylbutanoic acid, 3-methylbutanoic acid and 2-methylpropanoic acid) with fungal OTUs assigned to *Saccharomyces*. OTUs assigned to *Pichia, Candida* and *Aspergillus* (higher in controls) were negatively correlated with both *Saccharomyces* and BCFA (higher in active CD). *Saccharomyces* OTUs and BCFA were also positively correlated to many bacterial OTUs assigned to *Hafnia, Proteus, Lachnoclostridium, Lactobacillus, Proteus* etc. (Supplemental material 3, Figure S9-C).

**Figure 7.**
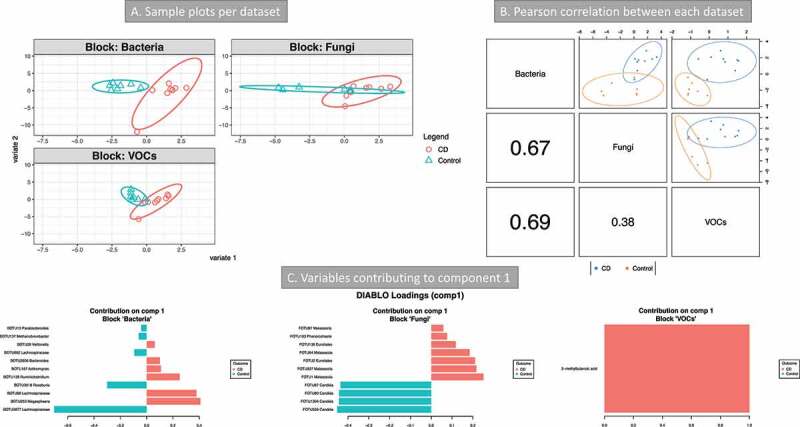
Integration of metabolomics (volatile organic compounds, VOCs) and metagenomics (bacterial and fungi) data (Crohn’s disease (CD) vs Controls). 16S and 18S rRNA datasets were from transverse colon biopsy samples, and VOCs from stool samples. Samples groups were CD (n = 9) vs Controls (n = 7). **A** three omics sample plots are shown separately. **B** Pearson’s correlation of the three data set for component 1. **C** variables contributing to component 1

A third integration was done by combining the micro- and myco-biome data from the transverse colon mucosa and VOCs from stool (Figure 7). CD patients were compared with controls. The lowest error rate (34%) was obtained with 1 component (Mahalanobis distance, Supplemental material 3, Figure S10). The bacterial community, followed by the metabolome, was the best dataset to discriminate between the two categories (Figure 7-A). The features from component 1 allowed the separation of the two groups for all three datasets (Figure 7-B). These features are presented in Figure 7-C. The majority of the bacterial OTUs found by this analysis were also implicated in taxa differential analysis and BV-STEP analysis. In particular, OTU2656 (*B. thetaiotamicron*, Figure 2-B). The fungal OTUs that were discriminant in component 1 space (Figure 7-C) were assigned to *Malassezia* and Eurotiales (increased in CD) and *Candida* (increased in control). Only one metabolite was included in this component: 3-methylbutanoic acid. Correlations of variables of different datasets are between 3-methylbutanoic acid and most of the bacterial OTUs increased in CD (positive correlations), including OTU2656 *Bacteroides. Candida* OTUs were negatively correlated to most of the bacterial OTUs increased in CD (including OTU2656 *B. thetaiotamicron*) and positively correlated to those increased in control (Supplemental material 2, Table S7).

## Discussion

This study is the first to investigate the microbiome and the mycobiome from stool and biopsies, and the fecal volatile metabolome in parallel in patients with Crohn’s disease and controls. These three datasets were integrated with a statistical procedure, that allows a consolidated view of multi-omics datasets. An optimized protocol^32^ was used for the investigation of fungal communities and standardized approaches to the microbiome^41^ and metabolome.^42^ We have clearly shown relationships between volatile metabolites involved in the degradation of amino acids and intestinal microbiota in CD and found fungal species (*Malassezia* and *Cyberlindnera*) increased in CD that may have a role to play in the disease (e.g. pathogenesis or they thrive in inflammation).

The British cohort enabled us to integrate three omics datasets (matching disease and controls); in addition, paired biopsies and feces were available from many donors. In stool, the most abundant genus was *Saccharomyces*, followed by *Candida*, as already reported.^43^ The limited amount of fungal material observed in the mucosa suggests the widespread presence of *Saccharomyces* in stool to the subjects’ dietary habits, as also recently suggested.^44^ However, our multi-omics analysis has shown *Saccharomyces* increased in active CD as compared to controls and is positively correlated with some of the metabolites. A potential role of *Saccharomyces* in CD has been proposed,^45^ although this theory was quickly opposed by Sendid *et al*.^46^ and by the observation of *S. cerevisiae* reduced in active IBD and that it could actually be beneficial.^12^

In stool (British cohort), a decrease of *Candida* abundance in CD patients was observed. This is in contrast to the current literature^11–13^ (and that observed in the Dutch cohort) that show *Candida* increased in IBD; we relate our observation to the small size of the cohort. However, we identified the negative correlation between *Candida* and *B. thetaiotamicron* (*Bt*), and we might speculate that the low abundance of *Candida* is related to the presence of *Bt* in the British cohort. Others have reported that *Bt* is able to prevent *Candida* colonization.^27^ A recent study also showed in a mouse model how *Bt* actually eliminates *Candida* species through enzymatic degradation.^47^ Evidence for inter-kingdom relationships is shown by the increase in *Ascomycota* and decrease of *Bacteroidetes* observed in cirrhotic patients that is predictive of hospitalization within 90 days.^48^ We believe that the inter-kingdom relationship between *Candida* and *Bacteroides* in IBD requires further exploration.

*Malassezia* was increased in the colonic mucosa but reduced in the stool samples of CD patients (British cohort). It was increased in CD patients from the Dutch cohort. Suhr *et al*. 2015^49^ reported that *Malassezia* is often found in stool and it is probably able to grow in the intestine.^50^ Further evidence comes from a recent study that showed *Malassezia* increased in the colonic mucosa of CD patients and that it exacerbated colitis in mice.^18^ Our observation of an increase of this yeast within the colon of CD patients supports the findings of this earlier study suggesting further research opportunities to clarify *Malassezia*’s role in CD.

In the Dutch cohort (Figure 5-D and supplemental material 6) *Candida* abundance increased with the disease (CD vs HC at baseline), meanwhile, *Saccharomyces* decreased, in agreement with earlier reports.^11–13^ Incorporating explanatory variables, that is, medication, has shown that variation in the fungal community at baseline can be explained by two medications: immunosuppressants and biologics. Patients who took immunosuppressants had significantly more *Candida* (OTU and genus level) and more *Malassezia* (genus level) (Figure 5-D2 and Supplemental material 6), although its abundance was low. Candidiasis are not uncommon in IBD patients treated with immunosuppressants,^51^ however, the mechanisms of how these classes of medications influence the fungal community is unknown. The temporal analysis of CD cohort has shown that the fungal community is less stable in CD patients and this was significant for patients who achieved remission. However, RA patients had a less variable community (lower beta-dispersion) when compared to patients achieving remission. We also observed that when they transitioned from remission to relapse, *Cyberlindnera* increased. An increase of *Cyberlindnera* during relapse has been previously reported.^16^

The analysis of the fungal community in the two cohorts has highlighted some discrepancies. Differences in diet, the use of healthy controls in the Dutch cohort, opposed to non-IBD individuals referred to gastroenterology clinic in the British cohort, and differences in the DNA extraction method may account for some of these. The British cohort also had fewer samples and the lack of separation between CD and controls in stool samples may be because the study was underpowered. However, even studies with a higher sampling struggled to find a separation between CD and controls, as was reported only when IBD and controls were compared.^12^

A dysbiosis of the bacterial community was observed both in the feces and biopsies of CD patients (British cohort), who were more dysbiotic than UC, as expected.^52^ The CD microbiome had lower alpha diversity estimates, formed a separate cluster in beta-diversity analysis, and was found to be more phylogenetically similar. Both the pathogenesis of CD and its distribution contributed to the variability in the microbiome. Disease distribution explains 15% of the diversity and there was a clear separation of CD patients with and without ileal involvement (Figure 1-B3). Other factors also influenced the microbiome composition (PERMANOVA analysis); the strongest predictor being patient ID, showing that the community was conserved in the mucosa and feces within an individual (R^2^ up to 79%). A similar pattern of samples originating from the same subject has been observed previously.^53^ The site of the biopsy was less relevant (circa 6%) (Figure 1-B4 and table in B2, Bray-Curtis distance). Extrinsic parameters (including clinical data) were also implicated in explaining the variability in the microbiome structure: in particular BMI, age, ASCA antibodies and some medications. However, it is difficult to confirm these influences as the CD patients were younger, leaner and more likely to have ASCA antibodies, as compared to UC and control subjects (Table 1). Therefore, these interpretations should be approached with caution. Drugs, including mesalamine and immunosuppressants^54^ can influence the gut microbiome.^55^ Mesalamine is taken by the majority of the patients with UC (15/20, Table 1) and we are unable to separate the influence of UC and mesalamine treatment. However, mesalamine has been shown to influence microbial metabolism and to reduce bacterial colonization of the mucosa.^56^

Taxa differential analysis has shown a reduction of *Faecalibacterium* in the colon and feces in CD, which is in agreement with other studies.^52^ Other microorganisms considered beneficial were also reduced in the colon and feces of CD patients: *Roseburia*^16^ and *Methanobrevibacter. Lachnoclostridium* was increased in CD, in every mucosa site and in the feces. *L. bolteae* (previously known as *Clostridium bolteae*), was proposed as a marker of dysbiosis related to the use of antibiotics^57^ and inflammation;^58^ however, it was decreased in CD pediatric patients.^8^ Further studies are indicated to clarify its role in dysbiosis and CD.

*Bacteroides* OTUs have shown mixed trends as some were increased in the mucosa of CD patients, although others were reduced. We found that *B. thetaiotamicron* (*Bt*) was increased in the colonic mucosa of CD patients (British cohort). *Bt* is crucial to the maturation of the gut immunity^59^ and microbiota,^60^ however, it can also have deleterious effects in the gut.^61–63^ Its interaction with *F. prausnitzii* (*Fp*) is particularly relevant in gut homeostasis; *Bt* prepares the gut environment for *Fp* to colonize, by reducing the redox potential;^60^ at the same time, *Fp* counterbalances and attenuates *Bt* effects in the gut.^60^ The decrease of *Fp* is likely to be one of the main events during dysbiosis and it is related to the increase in redox potential in the gut.^64,65^ Our observation of an increase of *Bt* and a simultaneous decrease of *Fp* in the colon of CD patients confirms that reduction of the latter actually causes the dysbiosis; a loss of *Fp* means that not only its anti-inflammatory action is lost,^66^ but also its counterbalancing effect on *Bt*. Moreover, the host susceptibility is likely to be a key factor in *Bt* impact in IBD.^61–63^

*B. vulgatus* was amongst the representative OTUs being part of the subsets that best correlated with the whole OTUs table (Supplemental material 2, Tables S2). OTUs assigned to this species were highly abundant in CD and controls. Both mechanistic and observational studies have shown that it causes inflammation only in susceptible hosts.^63,67^ Metatranscriptomic analysis shows that this is active in IBD patients.^68^

A final note on *Bacteroides* OTUs, many were part of the subsets obtained with the BV-STEP analysis, whose variability could be explained by calprotectin and increased in CD biopsies, showing a link between *Bacteroides* and inflammation, observed also by others.^69,70^

The analysis of VOCs in stool has shown an increase of metabolites associated with active CD,^5^ that is, 3,7-dimethylocta-1,6-dien-3-ol; nonanal; 2-methylpropyl butanoate; propyl 2-methyl propanoate; propyl propanoate. 3,7-dimethylocta-1,6-dien-3-ol is a monoterpene in culinary herbs and produced by some fungi,^37^ including Saccharomycetales yeasts.^39^ Nonanal is an aldehyde, and these molecules are often increased in CD: we reported this both in human CD^29^ and in a mouse model of IBD.^71^ This has been linked to inflammation and oxidative stress;^5^ in this context, these molecules are produced endogenously during a non-enzymatic lipid peroxidation.^72^

The observation of other compounds is less consistent with previously published literature, as these were actually proposed as discriminatory compounds for healthy subjects: indole; butanoic acid; 2-methylbutanoic acid; 3-methylbutanoic acid; 2-methylpropanoic acid; 2-pentanone.^5^ Other studies confirm our observation that indole increases in CD.^73,74^ Indole is produced during the catabolism of tryptophan by bacteria in the distal colon, in the presence of the free amino acid and absence of carbohydrates.^75,76^ SCFA (short chain fatty acid) are commonly produced in the colon during fiber degradation. In contrast, BCFA are exclusively produced by fermentation of branched-chained amino acids in the distal colon.^76^ Their increase in CD, observed here and by others,^74^ may be related to an increase in the fermentation of host amino acids obtained from luminal blood, mucins and inflamed tissue.^73^ Marchesi *et al*. linked the presence of amino acids (including the precursors of BCFA) in the colon of CD patients to malabsorption^77^ .

The integration of the mucosa associated micro- and myco-biome and VOCs from stool of the British cohort was the most informative of the three cases considered. It confirmed *Bt* to be a signature feature in the mucosa of CD patients. This was positively correlated to 3-methylbutanoic acid and negatively correlated with *Candida*. These analyses have also shown *Malassezia* as a diagnostic modality to discriminate between the CD and controls cohort.

The comparison of active CD patients and controls cohort has shown that OTUs assigned to Sacchromycetales yeasts were correlated with BCFA, suggesting that these strains may be metabolically active. *S. cerevisiae* can produce this molecule by valine degradation.^78^ 2-pentylfuran was also correlated with Saccharomycetales OTUs. Production of aldehydes can occur spontaneously during oxidative stress and may indicate inflammation rather than the activity of fungi;^79^ however, it may be consumed in some foods including soy milk^80^ and cheese production.^81^

Results of the comparison of all CD patients vs control were not included because the model gave an high error rate (close to 50%). This was likely related to the small sample size and to the high variability within the groups, due to many factors, such as the distribution and status of the disease.

To summarize, the use of Mixomics to integrate microbial with metabolomics data has shown that there is an increase of amino acid degradation in CD patients. Some of the VOCs were correlated with specific bacteria in addition to fungi in CD patients. This suggests that 1) fungi might be metabolically active in relapsing CD patients, 2) the degradation of amino acids is associated with CD and fungi might be involved in this. In the mucosa, this analysis confirmed that *Bt* and *Malassezia* are the representative features in CD. The role of *Bt* in controlling *Candida* colonization warrants further investigation.

There are some limitations in our study. The British cohort was relatively small which caused two issues: the analysis of the mycobiome did not support the hypothesis that it changes in diseased patients; the second issue was related to the multi-omics integration algorithm; the small cohort size gave a high BER that limits our ability to draw conclusive interpretations from our results. It is recently demonstrated that most of the mycobiome observed in the stool of healthy individuals is from food or the mouth,^44^ with the only exception of *C. albicans*, the only colonizer found so far in healthy individuals.^82^ This was also confirmed in our study, as we observed fungi only in a fraction of mucosa samples. In stool, the presence of fungi from the diet makes it difficult to discriminate between the mycobiota and fungi that are simply in transit. A recent study confirmed this discrepancy between mycobiome profiles from stool compared to biopsies, showing that *Debaryomyces* was prevalent (and viable) in the inflamed mucosa, but not in non-inflamed tissue or stool samples from the same individual.^20^ The use of a multi-omics approach may partially solve this issue, as the correlation of fungi with metabolites can be an indication of their activity, although a higher sample size is required in order to reduce the error rate and get more reliable correlations. The analysis of the transcriptome instead of the metagenome as a template for mycobiome analysis, coupled with metabolomics analysis, may overcome this issue altogether. Nonetheless, we have shown that an integrative approach such as the one considered in this study, informed by the latest in multivariate statistics, should be able to divulge underlying biological mechanisms that not otherwise possible by analyzing these datasets in isolation. Criticisms may rise by the use of 18S rRNA as target gene for the analysis of the mycobiome instead of the more commonly used ITS; however, we have already shown its efficacy in discriminating taxa relevant to the human gut.^32^

These data add a new layer of complexity to the microbial community in the intestine. Fungi reside in the mucosa and appear to interact with the bacterial community – especially *Bacteroides*. Mining these interactions is key to understanding the role of fungi on the disease and will lead to new potential therapies, i.e. manipulation of the microbiome^28^ informed by the individual micro- and myco-biota (personalized medicine). Our study did not show a direct link between fungi and CD, However, as discussed by Richard et al.^28^ only when specific opportunistic fungi are present, these appear to have a role in the severity of inflammation. Therefore, the use of antifungal and antibacterial agents should be used with care; and it is worth remembering that many drugs have off-target effects that act on microbes.^55,83,84^ Another aspect of our study was the integration of fungal and bacterial datasets with VOCs metabolomics data. We are aware that the small cohort did not allow to draw definite conclusions on the involvement of fungi in the production of fungal metabolites.^5^ However, we firmly believe that by carrying out integrated analysis on larger cohorts or on cohorts of patients with a confirmed fungal involvement, we will be able to show this direct link VOCs-fungi and to produce a very powerful diagnostic method that would greatly facilitate the application of personalized treatments.

In conclusion, our findings suggest that i) some fungal species, not only *Candida* but also *Malassezia* and *Cyberlindnera*, are likely to have a role in the pathogenesis and/or course of CD. Immunosuppression may increase *Candida* and *Malassezia* colonization in CD patients, ii) the low abundance of fungi in the mucosa means that fungi are not involved in all CD patients, iii) *Bacteroides* strains play a substantial role in CD and may modulate *Candida* colonization, and iv) amino acid degradation is associated with CD, and we identified bacterial and fungal species involved in their production or that are members of this trophic network.

## Methods

### Design and participants

This was an observational study of two cohorts from different geographic areas in Liverpool, UK, and Maastricht, The Netherlands. Patients recruited at the Royal Liverpool University Hospital (UK), between August 2015 and May 2017, were part of the European Union Seventh Framework Programme (EU-FP7) funded SysMedIBD (Systems Medicine of chronic Inflammatory Bowel Disease) Project (https://www.sysmedibd.eu/); they had CD or UC, or were non-IBD patients referred to the gastroenterology clinic who underwent diagnostic colonoscopy (controls). Demographic and clinical data including the diagnosis, disease phenotype (Montreal classification) and fecal calprotectin were collected. Donors from Maastricht were part of the Inflammatory Bowel Disease South Limburg Cohort (IBDSL) (The Netherlands):^85^ they included CD patients and healthy controls who were followed-up and sampled at least twice. All donors gave written informed consent before they were enrolled in the studies in accordance with ethical approval (reference 15/NW/0045 (Liverpool) and NL24572.018.08 (South Limburg)).

### Specimens

The British cohort stool samples were collected at home, stored chilled and brought to the hospital on the same day, or after overnight storage. Samples for DNA extraction were aliquoted immediately, transferred to the laboratory and stored at −80°C. Aliquots for VOCs analysis by gas chromatography mass spectrometry (GC-MS) were stored at −20°C. Biopsies of the ileum and colon were flash frozen in liquid nitrogen immediately, brought to the laboratory and stored at −80°C for later DNA extraction. Blood samples were collected in heparinized vials at 5 U/mL (Wockhardt UK Ltd; Wrexham; Wales): plasma was collected and stored at −20°C for later enzyme-linked immunosorbent assay (ELISA) for ASCA.

Only fecal samples were collected from the Dutch cohort: their collection and processing, including DNA extraction, performed with a modified protocol of the PSP stool DNA kit (Stratec), has been described in detail elsewhere:^34^ the extracted DNA was sent to the Liverpool laboratory for mycobiome studies.

### DNA extraction

Stool samples, from British subjects, were extracted using two kits to maximize extraction of fungal DNA, specifically a modified version of the PSP stool DNA kit (Stratec), and the QIAamp Fast DNA Stool mini kit (QIAGEN), details are described in Frau *et al*.^32^ DNA was extracted from the biopsies with the QIAamp *cador* Pathogen Mini Kit (Pretreatments T2, B1) (QIAGEN, Manchester, UK); it was quantified and normalized for downstream analysis as for feces.^32^

### Amplicons sequencing

Fungal 18S rRNA amplicons were sequenced for both cohorts, while bacterial 16S rRNA amplicons were sequenced only for the British cohort. A 2-step PCR universal tail tag dual index barcoding approach was used.^41^ Fungal amplicons were generated as previously described.^32^ For bacterial 16S rRNA gene (V4 region) sequencing, the method described by D’Amore *et al*.^41^ was used with a modified forward primer (Supplemental material 1, Table S1). Details are in the Supplemental material 1 (Supplementary Methods).

### VOCs

VOCs were analyzed following the method by Reade *at al*.^42^ Frozen stool samples were aliquoted (450–500 mg) and dispensed in 10 mL vials. VOCs were extracted from the headspace using a divinylbenzene-carboxen-polydimethylsiloxane solid-phase micro-extraction fiber (Sigma-Aldrich, Dorset, UK) and analyzed in a PerkinElmer Clarus 500 GC/MS quadrupole benchtop system (Beaconsfield, UK) fitted with a 60 m Zebron ZB-624 column (inner diameter 0.25 mm, length 60 m, film thickness 1.4 μm (Phenomenex, Macclesfield, UK)).^42^ Chromatograms were analyzed with Automated Deconvolution System (AMDIS) using the NIST mass spectral library to identify the peaks. This library included compounds related to fungi and CD markers.^5^ Statistical analysis was performed in Metaboanalyst,^86^ adjusting first the NA values to half of the minimum value for each compound and normalization by log transform (glog) and Pareto-scaling was performed, and reported using partial least squares discriminant analysis.

### ASCA ELISA

Anti-Saccharomyces cerevisiae antibody ELISA (IgG) was carried out with a commercial kit (EUROIMMUN cat. EV 2841–9601 G, Luebeck, Germany) using plasma samples which were analyzed in triplicate.

All experimental procedures were carried out following manufacture’s protocols and risk assessments were produced.

### Bioinformatics and statistical analysis

Details are in the Supplementary Methods (Supplemental material 1).

## Declarations

### Ethical statements

For specimens collected in Liverpool (UK), the ethical approval for the use of stool samples (SysMedIBD; EU-FP7 project, under grant agreement no. 305564) was granted by NRES Committee North West – Liverpool East, reference (15/NW/0045). For the specimens collected in the Netherlands (Inflammatory Bowel Disease South Limburg Cohort (IBDSL) (The Netherlands)), ethical approval was given by the Medical Ethics Committee of Maastricht University Medical Center and was executed according to the revised Declaration of Helsinki (59th General Assembly of WMA, Seoul, South Korea, Oct. 2008). The study has been registered in the Central Committee on Research Involving Human Subjects (CCMO) registry under file number NL24572.018.08.

### Availability of data and materials

The datasets of the sequencing raw reads supporting the conclusions of this article are available in the European Nucleotide Archive, under study accession numbers: PRJEB38969 (16S rRNA, stool and biopsy samples, British cohort), PRJEB29083 and PRJEB39119 (18S rRNA, stool samples, British cohort), PRJEB39016 (18S rRNA, biopsy samples, British cohort), PRJEB39063 (18S rRNA, stool samples, Dutch cohort).

## Supplementary Material

Supplemental Material
