# Supplementary material for: Inter-kingdom relationships in Crohn’s disease explored using a multi-omics approach
Source: Gut Microbes. 2021 Jul 9;13(1):1930871. doi: 10.1080/19490976.2021.1930871 (PMC8274447; doi:10.1080/19490976.2021.1930871)
Supplement: Supplemental Material [file KGMI_A_1930871_SM2617.zip › Supplementary information/Supplemental online material caption.docx]

# Supplemental online material

**Supplementary methods:** Supplemental material 1 (Supplemental_material_1_Supplementary_Methods.pdf).

**Supplementary results (tables)**: Supplemental material 2 (Supplemental_material_2_Supplementary_Results_Tables.pdf).

**Supplementary results (figures)**: Supplemental material 3 (Supplemental_material_3_Supplementary_Results_Figures.pdf).

**Table**: Supplemental material 4. Taxa differential analysis 1, results of the 16SrRNA data, British cohort (Supplemental_material_3_ Table_1.xlsx).

**Table**: Supplemental material 5. Taxa differential analysis 2, results of the 18SrRNA data, British cohort. (Supplemental_material_4_ Table_2.xlsx)

**Table**: Supplemental material 6. Taxa differential analysis 3, results of the 18SrRNA data, Dutch cohort (Supplemental_ material_5_ Table_3.xlsx).

**Table**: Supplemental material 7. Table of VOCs GC/MS raw data (Supplemental_material_7_VOCsRawData.xlsx).

**Table**: Supplemental material 8. Abundance tables at genus level for the mycobiome analysis (Supplemental_material_8_Table4.xlsx).
