# Supplementary material for: Inter-kingdom relationships in Crohn’s disease explored using a multi-omics approach
Source: Gut Microbes. 2021 Jul 9;13(1):1930871. doi: 10.1080/19490976.2021.1930871 (PMC8274447; doi:10.1080/19490976.2021.1930871)
Supplement: Supplemental Material [file KGMI_A_1930871_SM2617.zip › Supplementary information/Supplemental_material_1_Supplementary_Methods.pdf]

Inter-kingdom relationships in Crohn's disease explored using a multi-omics approach

Frau *et al.* Supplemental material 1

Supplementary Materials and Methods

*Table S1. Primers used for amplicon production during the first PCR round. The overhang + the specific primer for 18S rRNA and 16S rRNA were used. * this is a modification of the cited primer.*

Primer	***Sequence 5' to 3'***	***Marker***	***Ref***
Forward overhang	ACA CTC TTT CCC TAC ACG ACG CTC TTC CGA TCT	NA	(1)
Reverse overhang	GTG ACT GGA GTT CAG ACG TGT GCT CTT CCG ATC T		
FungiQuant-F (forward)	GGR AAA CTC ACC AGG TCCA G	18S rRNA	(2)
FungiQuant-R (reverse)	GSW CTA TCC CCA KCA CGA		
F515*	NNN NNG CMG CCG CGG TAA	16S rRNA	(3)
R806	GG ACT ACH VGG GTW TCT AAT		

Table S2 Primers (1) *used in the second PCR round to add the tags and sequencing primers to the amplicons. Combinations of the 8 forwards primers and 12 reverse primers were used to tag 96 samples (a full sequencing plate).*

Name	***Sequence***
FORWARD	
N501	AATGATACGGCGACCACCGAGATCTACACTAGATCGCACACTCTTTCCCTACACGACGCTC
N502	AATGATACGGCGACCACCGAGATCTACACCTCTCTATACACTCTTTCCCTACACGACGCTC
N503	AATGATACGGCGACCACCGAGATCTACACTATCCTCTACACTCTTTCCCTACACGACGCTC
N504	AATGATACGGCGACCACCGAGATCTACACAGAGTAGAACACTCTTTCCCTACACGACGCTC
N505	AATGATACGGCGACCACCGAGATCTACACGTAAGGAGACACTCTTTCCCTACACGACGCTC
N506	AATGATACGGCGACCACCGAGATCTACACACTGCATAACACTCTTTCCCTACACGACGCTC
N507	AATGATACGGCGACCACCGAGATCTACACAAGGAGTAACACTCTTTCCCTACACGACGCTC
N508	AATGATACGGCGACCACCGAGATCTACACCTAAGCCTACACTCTTTCCCTACACGACGCTC

REVERSE	
N701	CAAGCAGAAGACGGCATACGAGATTCGCCTTAGTGACTGGAGTTCAGACGTGTGCTC
N702	CAAGCAGAAGACGGCATACGAGATCTAGTACGGTGACTGGAGTTCAGACGTGTGCTC
N703	CAAGCAGAAGACGGCATACGAGATTTCTGCCTGTGACTGGAGTTCAGACGTGTGCTC
N704	CAAGCAGAAGACGGCATACGAGATGCTCAGGAGTGACTGGAGTTCAGACGTGTGCTC
N705	CAAGCAGAAGACGGCATACGAGATAGGAGTCCGTGACTGGAGTTCAGACGTGTGCTC
N706	CAAGCAGAAGACGGCATACGAGATCATGCCTAGTGACTGGAGTTCAGACGTGTGCTC
N707	CAAGCAGAAGACGGCATACGAGATGTAGAGAGGTGACTGGAGTTCAGACGTGTGCTC
N708	CAAGCAGAAGACGGCATACGAGATCCTCTCTGGTGACTGGAGTTCAGACGTGTGCTC
N709	CAAGCAGAAGACGGCATACGAGATAGCGTAGCGTGACTGGAGTTCAGACGTGTGCTC
N710	CAAGCAGAAGACGGCATACGAGATCAGCCTCGGTGACTGGAGTTCAGACGTGTGCTC
N711	CAAGCAGAAGACGGCATACGAGATTGCCTCTTGTGACTGGAGTTCAGACGTGTGCTC
N712	CAAGCAGAAGACGGCATACGAGATTCCTCTACGTGACTGGAGTTCAGACGTGTGCTC

Amplicons sequencing

In the first round PCR, the total reaction volume was 20 µL, with 0.02 U/µL Q5 High-Fidelity DNA Polymerase (New England Biolabs (NEB) Hitchin, UK), 1X Q5 Reaction Buffer (NEB, Hitchin, UK), 0.125 µM of each primer (HPLC grade, Integrated DNA Technology (IDT) Leuven, Belgium), 200 µM of dNTPs (NEB, Hitchin, UK) and Ultrapure DNAse/RNAse-free water (Life Technologies, Thermo Fisher Scientific, UK). 10 ng of DNA were used per reaction for stool samples and 80 ng of DNA were used for biopsy samples. Samples were amplified with the following program: 98°C for 30 seconds, then 15 (18S rRNA all amplicons, 16S rRNA biopsies) or 12 (16S rRNA British stool amplicons) cycles at 98°C for 10 seconds, 62°C (18S) / 65°C (16S) for 30 seconds and 72°C for 20 seconds, followed by a final extension at 72°C for 2 minutes. Amplicons were purified with the AxyPrep Mag PCR Clean-up kit (Axygen, Corning, Flintshire, UK) and eluted in 10 µL. The second PCR was carried out in 20 µL, using the same concentration of most reagents as the

first PCR, but with 0.25 µM of each index primer (Supplemental material 1, Table S2). The entire 10 µL volume of purified amplicons from the first PCR round was used as template. The amplification program was 98°C for 30 seconds, then 20 (16S rRNA biopsies and all 18S rRNA amplicons) or 15 (16S rRNA) cycles at 98°C for 10 seconds, 65°C for 30 seconds and 72°C for 20 seconds, followed by a final extension at 72°C for 2 minutes. Amplicons were purified, as above, and eluted in 25 µL. Positive controls (18S rRNA only, Supplemental material 2, Figure S12) and negative controls of the DNA extractions and of the PCR (no template) were also amplified and sequenced. PCRs were performed in triplicate and pooled before the last purification step. The pooling strategy, details of amplicons QC and Illumina MiSeq 2x250 bp sequencing can be found in Frau et al. (4).

Bioinformatics and Statistical analysis

Demultiplexing, adaptor and quality trimming (Cutadapt v 1.2.1(5) and Sickle v 1.2(6)) were performed by the Centre for Genomic Research (CGR), followed by primer trimming and error-correction with BayesHammer (7) (8) in SPAdes (v.3.7.0)(9), merging of paired-end reads with PEAR (v0.9.10)(10), and removal of PhiX with a custom script; this makes a BLAST search of PhiX sequence (GenBank gi9626372, max e-val <= 0.00001) and excludes from the dataset all the reads that match. Reads were filtered with the following parameters in bp: min pair length = 50, min length=200 (16S rRNA) and 300 (18S rRNA), max length=350 (16S rRNA) and 400 (18S rRNA), and samples with fewer than 1,000 reads were removed. Clustering was performed with SWARM 2.0 (11) (d=3). Chimeras were filtered with UCHIME (12) (ref mode, Greengenes(13) (gg_13_8) for 16S rRNA and SILVA (14) (SILVA_128) for 18S rRNA). Further analysis was done with MacQIIME (15) (v1.9.1); taxonomy assigned with BLAST (16); the reference databases was SILVA (14) (SILVA_123) for both 18S rRNA and 16S rRNA analysis. OTUs were filtered at a 0.05%

threshold (17). Alignment and phylogeny analyses were also carried out using MacQIIME (v1.9.1)(15), using default algorithms (PyNAST (18) and FastTree2 (19)). For fungal 18S rRNA reads, OTU tables were filtered to keep only Eukaryotic reads assigned to the Fungi kingdom. OTUs were considered contaminations when present in much higher abundance in the negative control and were excluded. When required, taxonomy assignment was also checked on BLASTN webtool (NCBI) (20), excluding uncultured/environmental sample sequences, and selecting match with 100% of coverage and identity.

Microbial data were analyzed with R (version 3.4.2) (21) using the scripts by the authors (22,23). The OTU table, phylogenetic tree and metadata were used for the statistical analysis. Vegan (24) was used for alpha and beta diversity analysis. For alpha diversity, data were rarified to the minimum library size and Shannon and Fisher alpha indices were calculated. For beta diversity, Bray-Curtis, unweighted and weighted UniFrac (25) distance measures were found using Phyloseq (26). Non-metric distance scaling was performed with the metamds() function. Additionally, 95% confidence interval of standard errors on NMDS plots were calculated with ordiellipse() function. For the Dutch cohort Principal Coordinates Analysis (PCoA) were used, instead of NMDS, to visualize beta-diversity cluster analysis. Vegan's adonis() function was used to perform analysis of variance (PERMANOVA) of sources of variations (groups in this study) against the distance matrices as mentioned above. Pair-wise ANOVA was calculated with aov(). Stability analysis performed on longitudinal data were made using the functions listed above: the change in alpha diversity (delta) was calculated for each individual and the average changes were subsequently compared between groups using pair-wise ANOVA, calculated with aov(). Similarly, beta-diversity was used to describe the stability of the community over time per individual. The within-subject beta-diversity for all subjects in the same group were then compared to other groups using aov() function (pair-wise ANOVA). Betadisperser() function of Vegan was used to assess

multivariate dispersion (variance), i.e. the distance of each sample from its group centroid (24). For taxa differential analysis DESeq2 (27) was used (DESeqDataSetFromMatrix()), we kept results with a significance value cut-off of 0.05 and fold-change of at least 2. Within taxa differential analysis, a random forest classifier was used to give ranking in terms of importance of each significant taxa; this was calculated using the randomForest() function (28). Two values were extracted with importance() function: Mean Decreased Accuracy (MDA) and Mean Decreased Gini (MDG). MDA indicates the loss of accuracy if that feature (taxa) was excluded from the model, whereas MGI reports the loss of purity. Taxa presented in the results had a total MDA 70-80%, unless the total MDA is <70-80%, in this case all the taxa were presented. Finally, subsets of OTUs that have maximum rank correlation with the whole OTU table were obtained with bioenv() function of Vegan (24). Evolview was used to visualize and customize phylogenetic trees (29).

We calculated the phylogenetic alpha diversity measures such as nearest taxa index (NTI) and net relatedness index (NRI) (30,31). This analysis helped determine whether the community structure is stochastic (i.e. driven by competition among taxa reflected by overdispersion of the phylogenetic tree) or deterministic (i.e. driven by strong environmental pressure with tight clustering). The NTI was calculated using mntd() and ses.mntd(), and the mean phylogenetic diversity (MPD) and NRI were calculated using mpd() and ses.mpd() functions from the R's picante package (32). NTI and NRI represent the negatives of the output from ses.mntd() and ses.mpd(), respectively. Additionally, they quantify the number of standard deviations that separate the observed values from the mean of the null distribution (999 randomisation using null.model-'richness' in the ses.mntd() and ses.mpd() functions and only considering taxa as either present or absent regardless of their relative abundance). NTI measures tip-level over/under dispersions (putting more emphasis on terminal clades to suggest "local" clustering) in phylogeny while NRI measures deeper dispersion (global

clustering). For both NTI and NRI, values greater than +2 indicate strong environmental pressure, and values less than -2 indicate strong competition among species as the driver of community structure. Whilst these boundaries are typically achieved in traditionally environmental datasets where gradients exist and species response is typically unimodal, for a constrained environment such as the host associated microbiome, these boundaries are rarely achieved, and instead should be interpreted in relative terms with an increasing value suggesting more environmental pressure.

Multi-omics analysis were made with Diablo (33), a framework of MixOmics (34). MixOmics uses supervised analysis to gather insights on features that allow discrimination between groups. Diablo specifically integrates data from different omics platforms, it is particularly useful to find features that correlate across the datasets analysed. Data were first normalized: Total Sum Scaling normalisation (TSS) followed by centered log-ratio (CLR) were used for microbial data (16S and 18S rRNA OTUs table), whereas Pareto-scaling was used for VOCs. Afterwards, the optimal number of components was assessed by fitting a model and cross-validating it with block.splsda() and perf() functions. An error rate (ER) and balanced error rate (BER) were obtained and the number of components with the lowest BER (mahalanobis distance) was selected and the final model obtained. Outputs of interest were computed using specific functions: plotDiablo() was used to obtain Pearson correlation between, and scatterplots of, different omics data set, one for each component; plotLoadings() shows the variables of each and their weight divided per omics. circosPlot() was used to compute and visualize the correlation between variables.

1. D'Amore R, Ijaz UZ, Schirmer M, Kenny JG, Gregory R, Darby AC, Shakya M, Podar M, Quince C, Hall N. A comprehensive benchmarking study of protocols and sequencing platforms for 16S rRNA community profiling. BMC Genomics 2016:17:1-20.

2. Liu CM, Kachur S, Dwan MG, Abraham AG, Aziz M, Hsueh PR, Huang YT, Busch JD, Lamit LJ, Gehring CA, Keim P. FungiQuant: A broad-coverage fungal quantitative real-time PCR assay. BMC Microbiol. 2012;12:1-11.
3. Bates ST, Berg-Lyons D, Caporaso JG, Walters WA, Knight R, Fierer N. Examining the global distribution of dominant archaeal populations in soil. ISME J. 2011;5:908–917.
4. Frau A, Kenny JG, Lenzi L, Campbell BJ, Ijaz UZ, Duckworth CA, Burkitt MD, Hall N, Anson J, Darby AC, Probert CS. DNA extraction and amplicon production strategies deeply influence the outcome of gut mycobiome studies. Sci. Rep. 2019;9:1-7.
5. Martin M. Cutadapt removes adapter sequences from high-throughput sequencing reads. EMBnet.journal 2011;17:10.
6. Joshi NA, Fass JN. Sickle: A sliding-window, adaptive, quality-based trimming tool for FastQ files. 2011;Available from: https://github.com/najoshi/sickle
7. Nikolenko SI, Korobeynikov AI, Alekseyev MA. BayesHammer: Bayesian clustering for error correction in single-cell sequencing. BMC Genomics 2013;14:1-11.
8. Schirmer M, Ijaz UZ, D'Amore R, Hall N, Sloan WT, Quince C. Insight into biases and sequencing errors for amplicon sequencing with the Illumina MiSeq platform. Nucleic Acids Res. 2015;43:e37–e37.
9. Nurk S, Bankevich A, Antipov D, Gurevich AA, Korobeynikov A, Lapidus A, Prjibelski AD, Pyshkin A, Sirotkin A, Sirotkin Y, Stepanauskas R. Assembling Single-Cell Genomes and Mini-Metagenomes From Chimeric MDA Products. J. Comput. Biol. 2013;20:714–737.
10. Zhang J, Kobert K, Flouri T, Stamatakis A. PEAR: a fast and accurate Illumina Paired-End reAd mergeR. Bioinformatics 2014;30:614–620.

11. Mahé F, Rognes T, Quince C, de Vargas C, Dunthorn M. Swarm v2: highly-scalable and high-resolution amplicon clustering. PeerJ 2015;3:e1420.
12. Edgar RC, Haas BJ, Clemente JC, Quince C, Knight R. UCHIME improves sensitivity and speed of chimera detection. Bioinformatics 2011;27:2194–2200.
13. McDonald D, Price MN, Goodrich J, Nawrocki EP, DeSantis TZ, Probst A, Andersen GL, Knight R, Hugenholtz P. An improved Greengenes taxonomy with explicit ranks for ecological and evolutionary analyses of bacteria and archaea. ISME J. 2012;6:610–618.
14. Quast C, Pruesse E, Yilmaz P, Gerken J, Schweer T, Yarza P, Peplies J, Glöckner FO. The SILVA ribosomal RNA gene database project: Improved data processing and web-based tools. Nucleic Acids Res. 2013;41:D590-6.
15. Caporaso JG, Kuczynski J, Stombaugh J, Bittinger K, Bushman FD, Costello EK, Fierer N, Pena AG, Goodrich JK, Gordon JI, Huttley GA. QIIME allows analysis of high-throughput community sequencing data. Nat. Methods 2010;7:335–336.
16. Edgar RC. Search and clustering orders of magnitude faster than BLAST. Bioinformatics 2010;26:2460–2461.
17. Bokulich NA, Subramanian S, Faith JJ, Gevers D, Gordon JI, Knight R, Mills DA, Caporaso JG. Quality-filtering vastly improves diversity estimates from Illumina amplicon sequencing. Nat. Methods 2013;10:57–59.
18. Caporaso JG, Bittinger K, Bushman FD, DeSantis TZ, Andersen GL, Knight R. PyNAST: a flexible tool for aligning sequences to a template alignment. Bioinformatics 2010;26:266–267.
19. Price MN, Dehal PS, Arkin AP. FastTree 2 - Approximately maximum-likelihood trees for large alignments. PLoS One 2010;5:e9490.
20. NCBI. Nucleotide blast (BLASTN). Available from: https://blast.ncbi.nlm.nih.gov

21. R Core Team. R: A Language and Environment for Statistical Computing. 2017.
22. Ijaz UZ. R scripts Microbiome Analysis. Available from: http://userweb.eng.gla.ac.uk/umer.ijaz/projects/microbiomeSeq_Tutorial.html
23. Ssekagiri A, Sloan WT, Ijaz UZ. microbiomeSeq: an R package for microbial community analysis in an environmental context. 2018.
24. Oksanen J, Blanchet FG, Friendly M, Kindt R, Legendre P, McGlinn D, Minchin PR, O'Hara RB, Simpson GL, Solymos P, et al. vegan: Community Ecology Package. 2020.
25. Lozupone C, Knight R. UniFrac: a new phylogenetic method for comparing microbial communities. Appl. Environ. Microbiol. 2005;71:8228–35.
26. McMurdie PJ, Holmes S. phyloseq: an R package for reproducible interactive analysis and graphics of microbiome census data. PLoS One 2013;8: e61217.
27. Love M, Huber W, Anders S. Moderated estimation of fold change and dispersion for RNA-seq data with DESeq2. Genome Biol. 2014;15:1-21.
28. Liaw A, Wiener M. Classification and Regression by randomForest. R News 2002;2/3:18–22.
29. He Z, Zhang H, Gao S, Lercher MJ, Chen WH, Hu S. Evolview v2: an online visualization and management tool for customized and annotated phylogenetic trees. Nucleic Acids Res. 2016;44:W236–W241.
30. Stegen JC, Lin X, Konopka AE, Fredrickson JK. Stochastic and deterministic assembly processes in subsurface microbial communities. ISME J. 2012;6:1653–1664.
31. Ijaz UZ, Sivaloganathan L, McKenna A, Richmond A, Kelly C, Linton M, Stratakos AC, Lavery U, Elmi A, Wren BW, Dorrell N. Comprehensive Longitudinal Microbiome Analysis of the Chicken Cecum Reveals a Shift From Competitive to Environmental Drivers and a Window of Opportunity for Campylobacter. Front.

Microbiol. 2018;9:2452.

32. Kembel SW, Cowan PD, Helmus MR, Cornwell WK, Morlon H, Ackerly DD, Blomberg SP, Webb CO. picante R tools for integrating phylogenies and ecology. Bioinformatics 2010; 26(11):1463-14644.

33. Singh A, Shannon CP, Gautier B, Rohart F, Vacher M, Tebbutt SJ, Lê Cao KA. DIABLO: from multi-omics assays to biomarker discovery, an integrative approach. bioRxiv 2018;067611.

34. Rohart F, Gautier B, Singh A, Rohart F, Gautier B, Singh A, Lê Cao KA. mixOmics: An R package for ‘omics feature selection and multiple data integration. PLOS Comput. Biol. 2017;13:e1005752.
