# Supplementary material for: Inter-kingdom relationships in Crohn’s disease explored using a multi-omics approach
Source: Gut Microbes. 2021 Jul 9;13(1):1930871. doi: 10.1080/19490976.2021.1930871 (PMC8274447; doi:10.1080/19490976.2021.1930871)
Supplement: Supplemental Material [file KGMI_A_1930871_SM2617.zip › Supplementary information/Supplemental_material_2_Supplementary_Results_Tables.pdf]

Inter-kingdom relationships in Crohn's disease explored using a multi-omics approach

Frau *et al.* Supplemental material 2

Supplementary Results (Tables)

Table S1 Results of PERMANOVA analysis of the bacterial community (British cohort). *This analysis was used to assess if any of the metadata (Age, BMI, Sex, Smoking, Calprotectin, ASCA and medications) explained the variations in the bacterial community between groups (R^2). PERMANOVA analysis was performed for all the predictors, taking each separately unless otherwise specified. Here only variables with significant results ($P \leq 0.05$) are presented (NS non-significant, . 0.1>p>0.5, * p<0.05, ** p< 0.01 and *** p< 0.001). ASCA, anti-*Saccharomyces cerevisiae *antibody BMI; Body Mass Index.*

Metadata	Site	Distance	Permanova
Age	Terminal Ileum	Bray-Curtis	NS
		UniFrac	R^2= 0.05 p = 0.027 *
		W. UniFrac	NS
	Transverse Colon	Bray-Curtis	R^2= 0.04 p = 0.078 .
		UniFrac	R^2= 0.06 p = 0.006 **
		W. UniFrac	NS
	Sigmoid Colon	Bray-Curtis	NS
		UniFrac	R^2= 0.05 p = 0.019 **
		W. UniFrac	NS
	Stool	Bray-Curtis	NS
		UniFrac	R^2= 0.04 p = 0.007 **
		W. UniFrac	R^2= 0.05 p = 0.006 **
BMI	Terminal Ileum	Bray-Curtis	R^2= 0.05 p = 0.084 .
		UniFrac	NS
		W. UniFrac	R^2= 0.09 p = 0.028 *
	Transverse Colon	Bray-Curtis	R^2= 0.05 p = 0.011 *
		UniFrac	R^2= 0.06 p = 0.015 *
		W. UniFrac	R^2= 0.11 p = 0.003 **
	Sigmoid Colon	Bray-Curtis	R^2= 0.05 p = 0.02 *
		UniFrac	R^2= 0.05 p = 0.073 .

		W. UniFrac	R^2= 0.1 p = 0.004 **
	Stool	Bray-Curtis	NS
		UniFrac	NS
		W. UniFrac	R^2= 0.04 p = 0.057 .
Sex	Terminal Ileum	Bray-Curtis	NS
		UniFrac	R^2= 0.05 p = 0.03 *
		W. UniFrac	NS
	Transverse Colon	Bray-Curtis	NS
		UniFrac	R^2= 0.04 p = 0.089 .
		W. UniFrac	NS
	Sigmoid Colon	Bray-Curtis	R^2= 0.04 p = 0.065 .
		UniFrac	R^2= 0.04 p = 0.082 .
		W. UniFrac	NS
ASCA	Terminal Ileum	Bray-Curtis	NS
		UniFrac	R^2= 0.06 p = 0.016 *
		W. UniFrac	NS
	Transverse Colon	Bray-Curtis	R^2= 0.06 p = 0.008 **
		UniFrac	R^2= 0.1 p = 0.001 ***
		W. UniFrac	R^2= 0.08 p = 0.015 *
	Sigmoid Colon	Bray-Curtis	R^2= 0.06 p = 0.007 **
		UniFrac	R^2= 0.1 p = 0.002 **
		W. UniFrac	R^2= 0.08 p = 0.016 *
	Stool	Bray-Curtis	NS
		UniFrac	R^2= 0.06 p = 0.001 ***
		W. UniFrac	R^2= 0.04 p = 0.073 .
Antibiotics	Sigmoid Colon	Bray-Curtis	NS
		UniFrac	R^2= 0.04 p = 0.027 *
		W. UniFrac	NS
	Stool	Bray-Curtis	NS
		UniFrac	R^2= 0.025 p = 0.064 .
		W. UniFrac	NS
Mesalamine	Terminal Ileum	Bray-Curtis	R^2= 0.07 p = 0.001 ***

		UniFrac	R^2= 0.05 p = 0.017 *
		W. UniFrac	R^2= 0.11 p = 0.003 **
	Transverse Colon	Bray-Curtis	R^2= 0.06 p = 0.001 ***
		UniFrac	NS
		W. UniFrac	R^2= 0.09 p = 0.003 **
	Sigmoid Colon	Bray-Curtis	R^2= 0.07 p = 0.001 ***
		UniFrac	R^2= 0.04 p = 0.095 .
		W. UniFrac	R^2= 0.1 p = 0.004 **
	Stool	Bray-Curtis	R^2= 0.026 p = 0.031 *
		UniFrac	NS
		W. UniFrac	R^2= 0.038 p = 0.033 *
Immunosuppressants	Stool	Bray-Curtis	R^2= 0.025 p = 0.043 *
		UniFrac	R^2= 0.03 p = 0.025 *
		W. UniFrac	NS

Table S2 BV-STEP routine results (Calprotectin). *Summary of OTUs (Operational taxonomy units) subsets that best rank correlate to the whole OTUs table (Bacterial 16S rRNA amplicons, British cohort). Permutational multivariate analysis of variance, PERMANOVA, (Adonis() function) was used to obtain the p values and the amount of variation explained by Calprotectin.*

**Stool**			
SUBSETS		Correlation with full OTU table (R)	PERMANOVA Calprotectin
Subset 1:	OTU2191 + OTU142 + OTU50 + OTU1804 + OTU19 + OTU70 + OTU721	0.692	R^2 = 0.10 (p = 0.002) (**)
Subset 2:	OTU2191 + OTU142 + OTU1574 + OTU50 + OTU1804 + OTU19 + OTU70 + OTU721	0.69	R^2 = 0.072 (p = 0.009) (**)
Subset 3:	OTU2191 + OTU142 + OTU50 + OTU1804 + OTU19 + OTU721	0.69	R^2 = 0.11 (p = 0.003) (**)

Subset 4:	OTU2191 + OTU142 + OTU50 + OTU19 + OTU721	0.674	R^2 = 0.12 (p = 0.001) (***)
Subset 5:	OTU2191 + OTU142 + OTU50 + OTU19	0.66	R^2 = 0.12 (p = 0.002) (**)
Subset 6:	OTU2191 + OTU142 + OTU50	0.64	R^2 = 0.16 (p = 0.001) (***)
Subset 7:	OTU142 + OTU50	0.6	R^2 = 0.16 (p = 0.001) (***)
OTU2191:Bacteria;Bacteroidetes;Bacteroidia;Bacteroidales;Bacteroidaceae;Bacteroides; OTU142:Bacteria;Firmicutes;Clostridia;Clostridiales;Ruminococcaceae;Flavonifractor; OTU50:Bacteria;Firmicutes;Clostridia;Clostridiales;Ruminococcaceae;Faecalibacterium; OTU1804:Bacteria;Bacteroidetes;Bacteroidia;Bacteroidales;Bacteroidaceae;Bacteroides; OTU19:Bacteria;Bacteroidetes;Bacteroidia;Bacteroidales;Rikenellaceae;Alistipes; OTU70:Bacteria;Firmicutes;Clostridia;Clostridiales;Ruminococcaceae;Subdoligranulum; OTU721:Bacteria;Firmicutes;Clostridia;Clostridiales;Ruminococcaceae;Faecalibacterium; OTU1574:Bacteria;Bacteroidetes;Bacteroidia;Bacteroidales;Prevotellaceae;Prevotella 9			
**Terminal Ileum**			
SUBSETS		Correlation with full OTU table (R)	PERMANOVA Calprotectin
Subset 1:	OTU2191 + OTU75 + OTU86 + OTU16 + OTU247 + OTU2879 + OTU2656 + OTU721 + OTU28 + OTU80	0.75	R^2 = 0.14 (p = 0.013) (*)
Subset 2:	OTU2191 + OTU75 + OTU86 + OTU16 + OTU2879 + OTU2656 + OTU721 + OTU28 + OTU80	0.75	R^2 = 0.16 (p = 0.014) (*)
Subset 3:	OTU2191 + OTU75 + OTU86 + OTU128 + OTU16 + OTU247 + OTU2879 + OTU2656 + OTU721 + OTU28 + OTU80	0.74	R^2 = 0.16 (p = 0.011) (*)
Subset 4:	OTU2191 + OTU86 + OTU16 + OTU2879 + OTU2656 + OTU721 + OTU28 + OTU80	0.74	R^2 = 0.16 (p = 0.011) (*)

Subset 5:	OTU2191 + OTU75 + OTU86 + OTU16 + OTU2879 + OTU2656 + OTU721 + OTU28	0.73	R^2 = 0.16 (p = 0.004) (**)
Subset 6:	OTU2191 + OTU75 + OTU86 + OTU16 + OTU2879 + OTU2656 + OTU721	0.735	R^2 = 0.16 (p = 0.007) (**)
Subset 7:	OTU2191 + OTU86 + OTU16 + OTU2879 + OTU2656 + OTU721	0.73	R^2 = 0.16 (p = 0.007) (**)
Subset 8:	OTU2191 + OTU86 + OTU2879 + OTU2656 + OTU721	0.72	R^2 = 0.17 (p = 0.004) (**)
Subset 9:	OTU2191 + OTU2879 + OTU2656 + OTU721	0.71	R^2 = 0.16 (p = 0.006) (**)
Subset 10:	OTU2191 + OTU2656 + OTU721	0.70	R^2 = 0.16 (p = 0.006) (**)
Subset 11:	OTU2191 + OTU721	0.65	R^2 = 0.14 (p = 0.064) (.)

OTU2191:Bacteria;Bacteroidetes;Bacteroidia;Bacteroidales;Bacteroidaceae;Bacteroides;

OTU75:Bacteria;Firmicutes;Clostridia;Clostridiales;Lachnospiraceae;Lachnospiraceae ND3007 group;

OTU86:Bacteria;Firmicutes;Clostridia;Clostridiales;Lachnospiraceae;Dorea;Dorea formicigenerans ATCC 27755

OTU16:Bacteria;Firmicutes;Clostridia;Clostridiales;Lachnospiraceae;Roseburia;

OTU247:Bacteria;Firmicutes;Clostridia;Clostridiales;Lachnospiraceae;Lachnoclostridium;Ruminococcus torques ATCC 27756

OTU2879:Bacteria;Firmicutes;Clostridia;Clostridiales;Lachnospiraceae;Lachnoclostridium;

OTU2656:Bacteria;Bacteroidetes;Bacteroidia;Bacteroidales;Bacteroidaceae;Bacteroides;

OTU721:Bacteria;Firmicutes;Clostridia;Clostridiales;Ruminococcaceae;Faecalibacterium;

OTU28:Bacteria;Bacteroidetes;Bacteroidia;Bacteroidales;Bacteroidaceae;Bacteroides;

OTU80:Bacteria;Firmicutes;Clostridia;Clostridiales;Lachnospiraceae;Blautia;

OTU128:Bacteria;Firmicutes;Clostridia;Clostridiales;Ruminococcaceae;Ruminiclostridium 5

Sigmoid Colon

SUBSETS		Correlation with full OTU table (R)	PERMANOVA Calprotectin
Subset 1:	OTU2191 + OTU128 + OTU43 + OTU2279 + OTU360 + OTU24 + OTU28	0.58	$R^2 = 0.13$ ($p = 0.008$) (**)
Subset 2:	OTU2191 + OTU128 + OTU43 + OTU2279 + OTU360 + OTU2 + OTU24 + OTU28	0.58	$R^2 = 0.13$ ($p = 0.006$) (**)
Subset 3:	OTU2191 + OTU43 + OTU2279 + OTU360 + OTU24 + OTU28	0.57	$R^2 = 0.13$ ($p = 0.009$) (**)
Subset 4:	OTU2191 + OTU2279 + OTU360 + OTU24 + OTU28	0.56	$R^2 = 0.11$ ($p = 0.031$) (*)
Subset 5:	OTU2191 + OTU2279 + OTU360 + OTU28	0.54	$R^2 = 0.11$ ($p = 0.072$) (.)
Subset 6:	OTU2191 + OTU360 + OTU28	0.50	$R^2 = 0.18$ ($p = 0.02$) (*)
Subset 7:	OTU360 + OTU28	0.47	$R^2 = 0.2$ ($p = 0.006$) (**)
OTU2191:Bacteria;Bacteroidetes;Bacteroidia;Bacteroidales;Bacteroidaceae;Bacteroides;<br>OTU128:Bacteria;Firmicutes;Clostridia;Clostridiales;Ruminococcaceae;Ruminiclostridium 5;<br>OTU43:Bacteria;Proteobacteria;Gammaproteobacteria;Enterobacteriales;Enterobacteriaceae;Escherichia-Shigella;<br>OTU2279:Bacteria;Bacteroidetes;Bacteroidia;Bacteroidales;Bacteroidaceae;Bacteroides;<br>OTU360:Bacteria;Firmicutes;Clostridia;Clostridiales;Ruminococcaceae;Subdoligranulum;<br>OTU24:Bacteria;Bacteroidetes;Bacteroidia;Bacteroidales;Porphyromonadaceae;Odoribacter;<br>OTU28:Bacteria;Bacteroidetes;Bacteroidia;Bacteroidales;Bacteroidaceae;Bacteroides;<br>OTU2:Bacteria;Bacteroidetes;Bacteroidia;Bacteroidales;Bacteroidaceae;Bacteroides;			

Table S3 Results of PERMANOVA analysis of the fungal community (British cohort). *This analysis was used to assess if any of the metadata (Age, BMI, Sex, Smoking, Calprotectin, ASCA and medications) explained the variations in the fungal community between groups (R^2). When all biopsy sites were analysed, patient ID was also added to the analysis along with*

*the parameter analysed. Here only variables with significant results (P ≤0.05) are presented (NS non-significant, . 0.1>p>0.5, * p<0.05, ** p< 0.01 and *** p< 0.001). ASCA, anti-Saccharomyces cerevisiae antibody. BMI, Body Mass Index. PERMANOVA Permutational multivariate analysis of variance.*

Metadata	Site	Distance	Permanova
Patient	All biopsy sites	Bray-Curtis	R2= 0.58 p = 0.001 ***
		UniFrac	R2= 0.75 p = 0.001 ***
		W. UniFrac	R2= 0.58 p = 0.014 *
Age	All biopsy sites	Bray-Curtis	NS
		UniFrac	R2= 0.03 p = 0.001 ***
		W. UniFrac	R2= 0.07 p = 0.09 .
	Sigmoid Colon	Bray-Curtis	NS
		UniFrac	NS
		W. UniFrac	R^2= 0.07 p = 0.09 .
Sex	All biopsy sites	Bray-Curtis	R^2= 0.02 p = 0.021 *
		UniFrac	R^2= 0.02 p = 0.006 **
		W. UniFrac	NS
	Terminal Ileum	Bray-Curtis	R^2= 0.06 p = 0.093 .
		UniFrac	NS
		W. UniFrac	NS
Smoking	All biopsy sites	Bray-Curtis	NS
		UniFrac	R^2= 0.025 p = 0.001 ***
		W. UniFrac	NS
	Transverse Colon	Bray-Curtis	R^2= 0.06 p = 0.003 *
		UniFrac	NS
		W. UniFrac	NS
Calprotectin	All biopsy sites	Bray-Curtis	R^2= 0.03 p = 0.001 ***
		UniFrac	R^2= 0.035 p = 0.001 ***
		W. UniFrac	R^2= 0.02 p = 0.091 .
	Terminal Ileum	Bray-Curtis	NS
		UniFrac	R^2= 0.07 p = 0.052 .
		W. UniFrac	R^2= 0.08 p = 0.098 .
	Transverse Colon	Bray-Curtis	NS

		UniFrac	R^2= 0.07 p = 0.054 .
		W. UniFrac	R^2= 0.08 p = 0.099 .
Antibiotics	All biopsy sites	Bray-Curtis	NS
		UniFrac	R^2= 0.02 p = 0.003 **
		W. UniFrac	NS
	Terminal Ileum	Bray-Curtis	NS
		UniFrac	R^2= 0.07 p = 0.054 .
		W. UniFrac	R^2= 0.09 p = 0.064 .
Mesalamine	All biopsy sites	Bray-Curtis	NS
		UniFrac	R^2= 0.03 p = 0.001 ***
		W. UniFrac	NS
Steroid	All biopsy sites	Bray-Curtis	R^2= 0.015 p = 0.061 .
		UniFrac	NS
		W. UniFrac	NS
Biologics	All biopsy sites	Bray-Curtis	R^2= 0.02 p = 0.013 *
		UniFrac	R^2= 0.015 p = 0.01 **
		W. UniFrac	NS
Proton pump inhibitor	All biopsy sites	Bray-Curtis	NS
		UniFrac	R^2= 0.02 p = 0.003 **
		W. UniFrac	NS
Selective Serotinin Reuptake Inhibitor	All biopsy sites	Bray-Curtis	NS
		UniFrac	R^2= 0.01 p = 0.042 *
		W. UniFrac	NS

Table S4 Results of PERMANOVA analysis of the fungal community (Dutch cohort). *This analysis was used to assess if any of the metadata (Age, Sex, Smoking, Calprotectin and medications) explained the variations in the fungal community between groups (R^2). Here only variables with significant results ($P \leq 0.05$) are presented (NS non-significant, . 0.1>p>0.5, * p<0.05, ** p< 0.01 and *** p< 0.001). PERMANOVA Permutational multivariate analysis of variance.*

Metadata	Distance	PERMANOVA
Calprotectin	Bray-Curtis	R^2= 0.06 p = 0.075 .
	UniFrac	R^2= 0.05 p = 0.099 .

	W. UniFrac	NS
Biologics	Bray-Curtis	R^2= 0.12 p = 0.005 **
	UniFrac	R^2= 0.06 p = 0.005 **
	W. UniFrac	R^2= 0.19 p = 0.006 **
Immunosuppressants	Bray-Curtis	NS
	UniFrac	R^2= 0.09 p = 0.005 **
	W. UniFrac	R^2= 0.08 p = 0.066 .

Table S5 Circo plot correlations between relevant variables of the DIABLO model. *The integration was of the micro and mycobiome of mucosa samples from the transverse colon stool volatile organic compounds (VOCs) and comparing Crohn's disease (CD) (red) vs Controls (blue).*

Var1	Var2	value
BOTU128 Ruminiclostridium	BOTU2656 Bacteroides	0.67184853
BOTU128 Ruminiclostridium	BOTU253 Megasphaera	0.6182323
BOTU253 Megasphaera	BOTU167 Actinomyces	0.64683985
BOTU26 Veillonella	BOTU66 Lachnospiraceae	0.62612583
BOTU253 Megasphaera	BOTU66 Lachnospiraceae	0.64680743
BOTU167 Actinomyces	BOTU66 Lachnospiraceae	0.65144593
BOTU128 Ruminiclostridium	3-methylbutanoic acid	0.77443532
BOTU2656 Bacteroides	3-methylbutanoic acid	0.75516695
FOTU64 Malassezia	3-methylbutanoic acid	0.63673019
FOTU927	3-methylbutanoic acid	0.67248258
BOTU26 Veillonella	3-methylbutanoic acid	0.67346487
BOTU253 Megasphaera	3-methylbutanoic acid	0.78922557
BOTU167 Actinomyces	3-methylbutanoic acid	0.77410107
BOTU66 Lachnospiraceae	3-methylbutanoic acid	0.79791443
3-methylbutanoic acid	BOTU15 Parabacteroides	-0.6451291

BOTU253 Megasphaera	BOTU3877 Lachnospiraceae Lachnospiraceae	-0.6120413
BOTU167 Actinomyces	BOTU3877 Lachnospiraceae	-0.6156556
BOTU66 Lachnospiraceae	BOTU3877 Lachnospiraceae	-0.6799546
3-methylbutanoic acid	BOTU3877 Lachnospiraceae	-0.7979655
BOTU128 Ruminiclostridium	FOTU559 Candida	-0.655044
BOTU2656 Bacteroides	FOTU559 Candida	-0.6753159
FOTU64 Malassezia	FOTU559 Candida	-0.6037393
FOTU927	FOTU559 Candida	-0.6381975
BOTU26 Veillonella	FOTU559 Candida	-0.6767184
BOTU253 Megasphaera	FOTU559 Candida	-0.7468548
BOTU167 Actinomyces	FOTU559 Candida	-0.739452
BOTU66 Lachnospiraceae	FOTU559 Candida	-0.779355
3-methylbutanoic acid	FOTU559 Candida	-0.9291287
BOTU15 Parabacteroides	FOTU559 Candida	0.63263311
BOTU3877 Lachnospiraceae	FOTU559 Candida	0.76304198
BOTU128 Ruminiclostridium	FOTU67 Candida	-0.6736949
BOTU2656 Bacteroides	FOTU67 Candida	-0.705494
BOTU253 Megasphaera	FOTU67 Candida	-0.6902124
BOTU167 Actinomyces	FOTU67 Candida	-0.6816266
BOTU66 Lachnospiraceae	FOTU67 Candida	-0.7186516
3-methylbutanoic acid	FOTU67 Candida	-0.9234734
BOTU3877 Lachnospiraceae	FOTU67 Candida	0.76541145
FOTU559 Candida	FOTU67 Candida	0.85044264
BOTU128 Ruminiclostridium	FOTU1304 Candida	-0.741465
BOTU2656 Bacteroides	FOTU1304 Candida	-0.737069
BOTU253 Megasphaera	FOTU1304 Candida	-0.7020936

BOTU167 Actinomyces	FOTU1304 Candida	-0.6822832
BOTU66 Lachnospiraceae	FOTU1304 Candida	-0.6848208
3-methylbutanoic acid	FOTU1304 Candida	-0.9224564
BOTU3877 Lachnospiraceae	FOTU1304 Candida	0.73106221
FOTU559 Candida	FOTU1304 Candida	0.83625183
FOTU67 Candida	FOTU1304 Candida	0.8875214
BOTU128 Ruminiclostridium	FOTU80 Candida	-0.7544762
BOTU2656 Bacteroides	FOTU80 Candida	-0.7371709
BOTU253 Megasphaera	FOTU80 Candida	-0.7047308
BOTU167 Actinomyces	FOTU80 Candida	-0.683586
BOTU66 Lachnospiraceae	FOTU80 Candida	-0.6826604
3-methylbutanoic acid	FOTU80 Candida	-0.9212677
BOTU3877 Lachnospiraceae	FOTU80 Candida	0.72358134
FOTU559 Candida	FOTU80 Candida	0.83372821
FOTU67 Candida	FOTU80 Candida	0.87999617
FOTU1304 Candida	FOTU80 Candida	0.89210456
3-methylbutanoic acid	BOTU692 Lachnospiraceae	-0.6743365
FOTU559 Candida	BOTU692 Lachnospiraceae	0.6116306
FOTU67 Candida	BOTU692 Lachnospiraceae	0.68933035
FOTU1304 Candida	BOTU692 Lachnospiraceae	0.70274376
FOTU80 Candida	BOTU692 Lachnospiraceae	0.69190568
BOTU128 Ruminiclostridium	BOTU137 Methanobrevibacter	-0.7138591
3-methylbutanoic acid	BOTU137 Methanobrevibacter	-0.6162924
BOTU128 Ruminiclostridium	BOTU3618 Roseburia	-0.7554631
BOTU2656 Bacteroides	BOTU3618 Roseburia	-0.6697545
3-methylbutanoic acid	BOTU3618 Roseburia	-0.7800805
FOTU559 Candida	BOTU3618 Roseburia	0.66420865

FOTU67 Candida	BOTU3618 Roseburia	0.76849391
FOTU1304 Candida	BOTU3618 Roseburia	0.79841949
FOTU80 Candida	BOTU3618 Roseburia	0.79753666
BOTU692 Lachnospiraceae	BOTU3618 Roseburia	0.61339762
BOTU137 Methanobrevibacter	BOTU3618 Roseburia	0.61138818
