# Supplementary material for: Inter-kingdom relationships in Crohn’s disease explored using a multi-omics approach
Source: Gut Microbes. 2021 Jul 9;13(1):1930871. doi: 10.1080/19490976.2021.1930871 (PMC8274447; doi:10.1080/19490976.2021.1930871)
Supplement: Supplemental Material [file KGMI_A_1930871_SM2617.zip › Supplementary information/Supplemental_material_3_Supplementary_Results_Figures.pdf]

Inter-kingdom relationships in Crohn's disease explored using a multi-omics approach

Frau *et al.* Supplemental material 3

Supplementary Results (Figures)

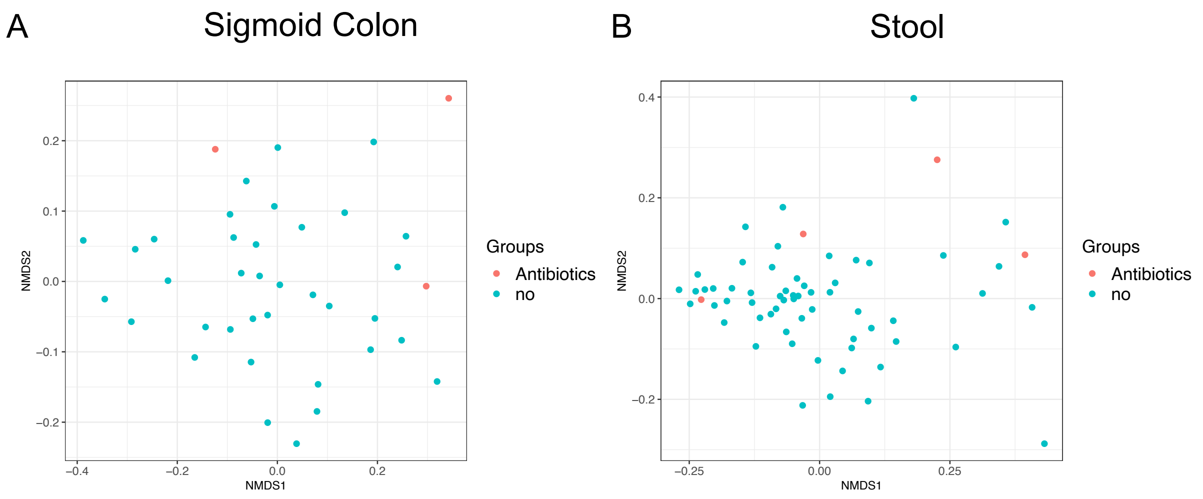

Figure S1 Nonmetric distance scaling (NMDS) showing clustering of samples (Bacterial 16S rRNA amplicons, British cohort). *These charts represent a combination of specimen type / site and distance metric for which a significant p value was obtained (Permutational multivariate analysis of variance, PERMANOVA) when clustering the samples according to the use of antibiotics (Supplementary material 2, Table S1). A) All sigmoid colon samples (n = 39), clustering was calculated using unweighted Unifrac Distance. B) all stool samples (n=63), clustering was calculated with weighted UniFrac.*

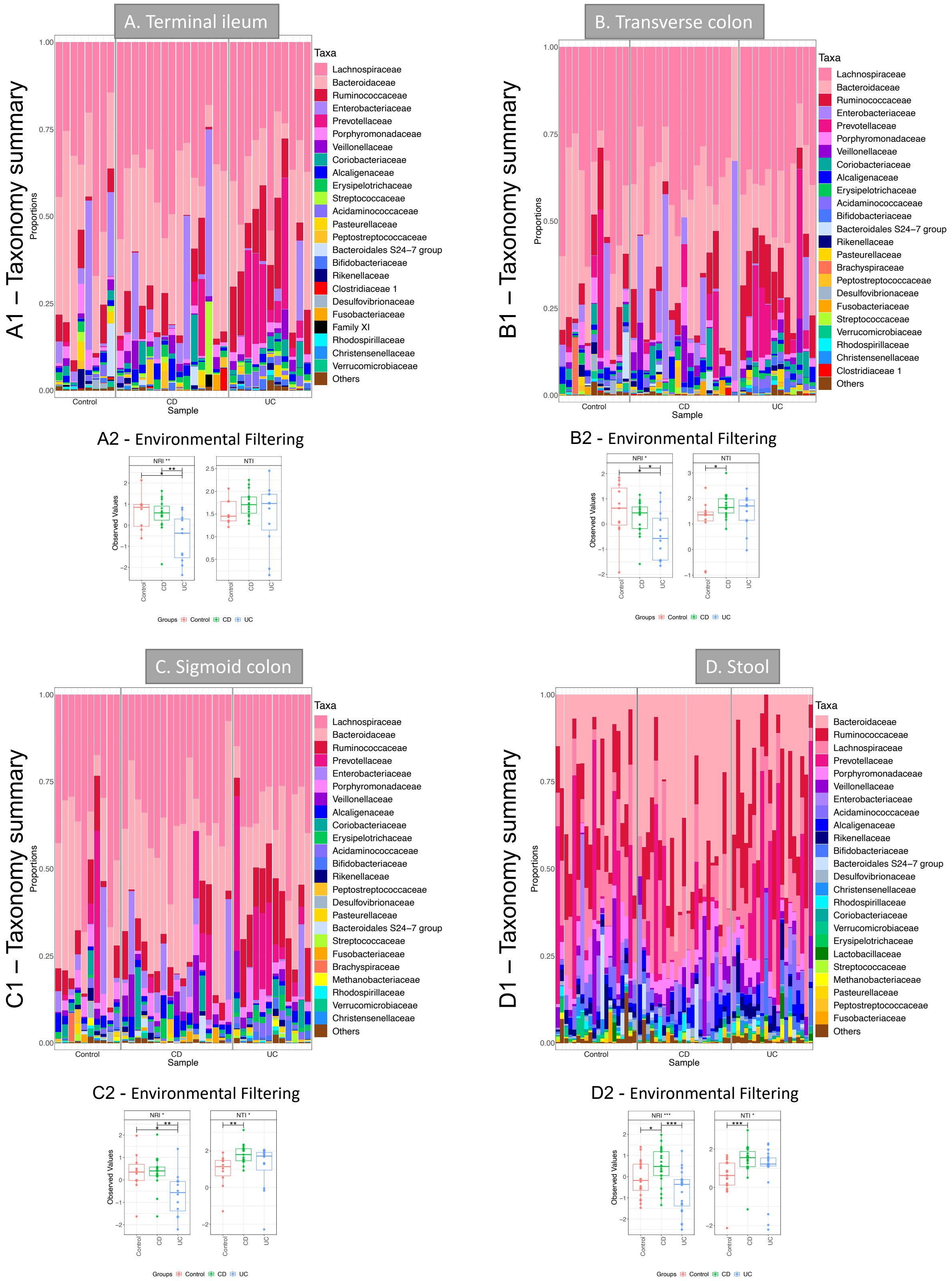

Figure S2 Taxonomy summary, taxa differential analysis and environmental filtering for the bacterial community (British cohort). *A: Terminal Ileum samples; B: Transverse colon samples; C: sigmoid colon samples; D: stool results samples. In A1, B1, C1 and D1 taxa summary at family level (dominant taxa) are presented. In A2, B2, C2 and D2 environmental filtering (weighted). Positive NTI (Nearest taxon index) indicates higher phylogenetic clustering and it is*

*calculated using the average branch length to the nearest co-occurring taxon, it is therefore more sensitive to patterns concerning the tips of the phylogenetic tree. Significant results are presented with an * (* p<0.05, ** p< 0.01 and *** p< 0.001).*

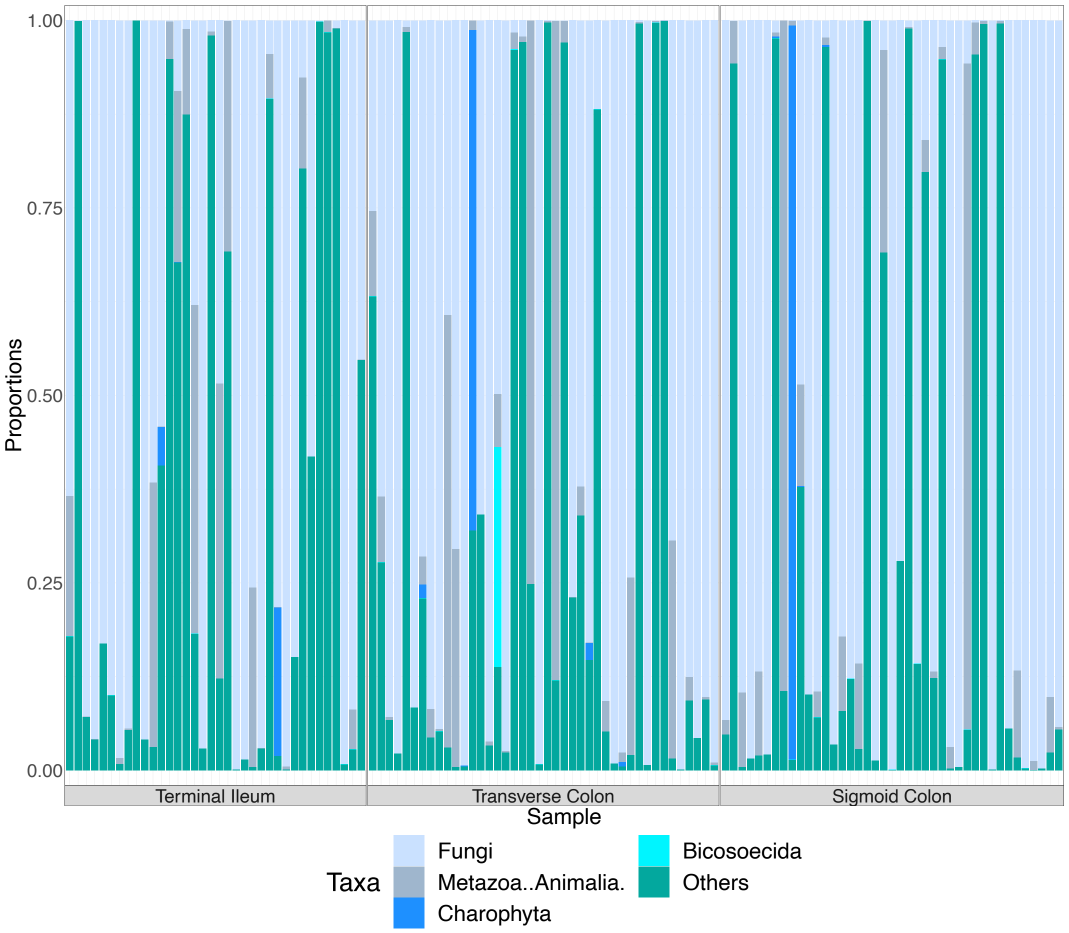

Figure S3 Taxa summary at kingdom level of biopsy samples (fungal 18S rRNA, British cohort). *Samples were from the terminal ileum, transverse colon and sigmoid colon (n = 119).*

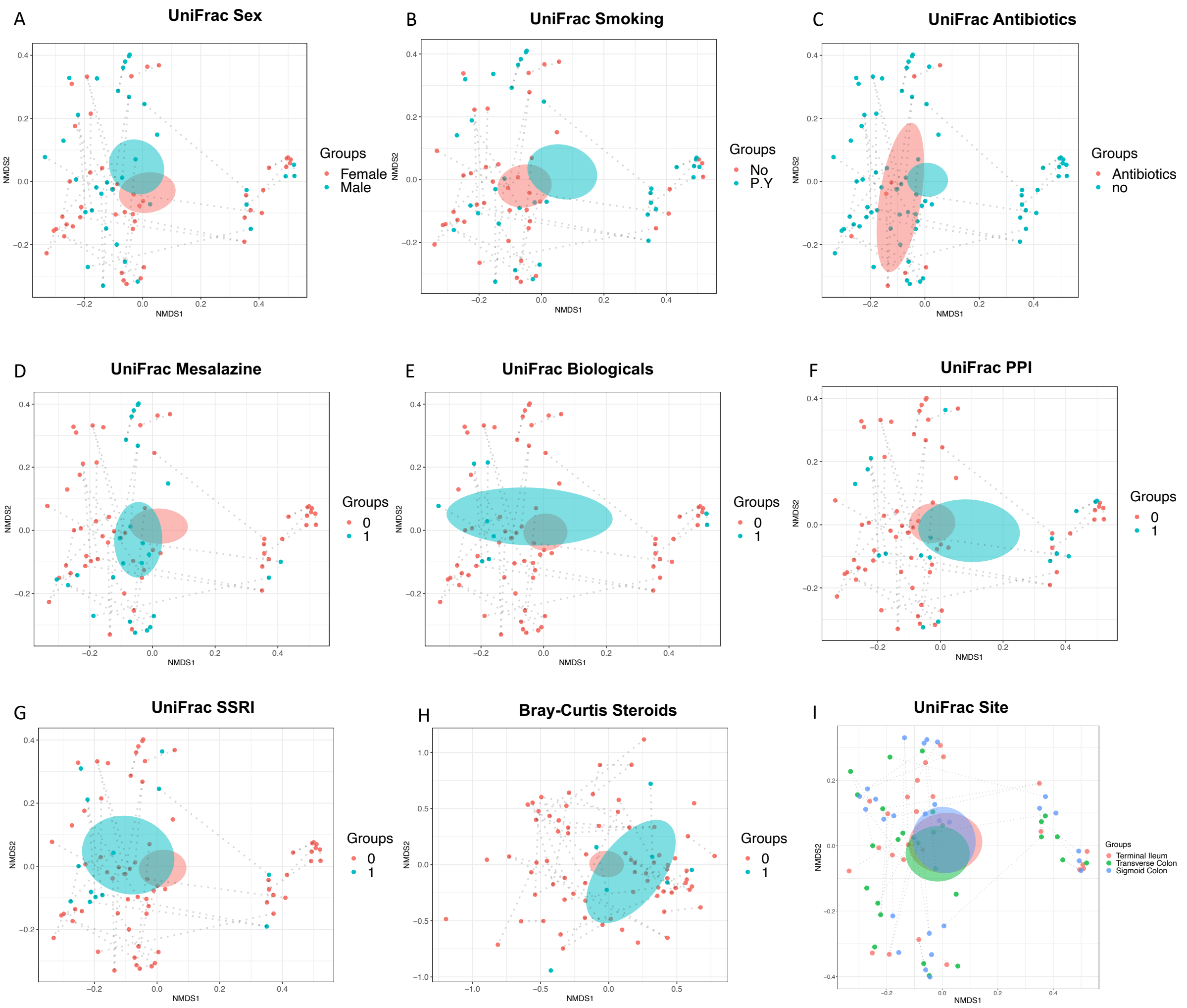

Figure S4 Nonmetric distance scaling (NMDS) showing clustering of biopsy samples (Fungal community, British cohort). *These charts represent a combination of specimen type and distance metric for which a significant p value was obtained (Permutational multivariate analysis of variance, PERMANOVA) when clustering the samples according to metadata (Table S4). Distance and metadata are indicated above each chart. Dotted lines link biopsy samples coming from the same individual. As a reference grouping according to biopsy site is in box I. P.Y. = previous or current smoker. PPI = proton pump inhibitor. SSRI = Selective Serotinin Reuptake Inhibitor.*

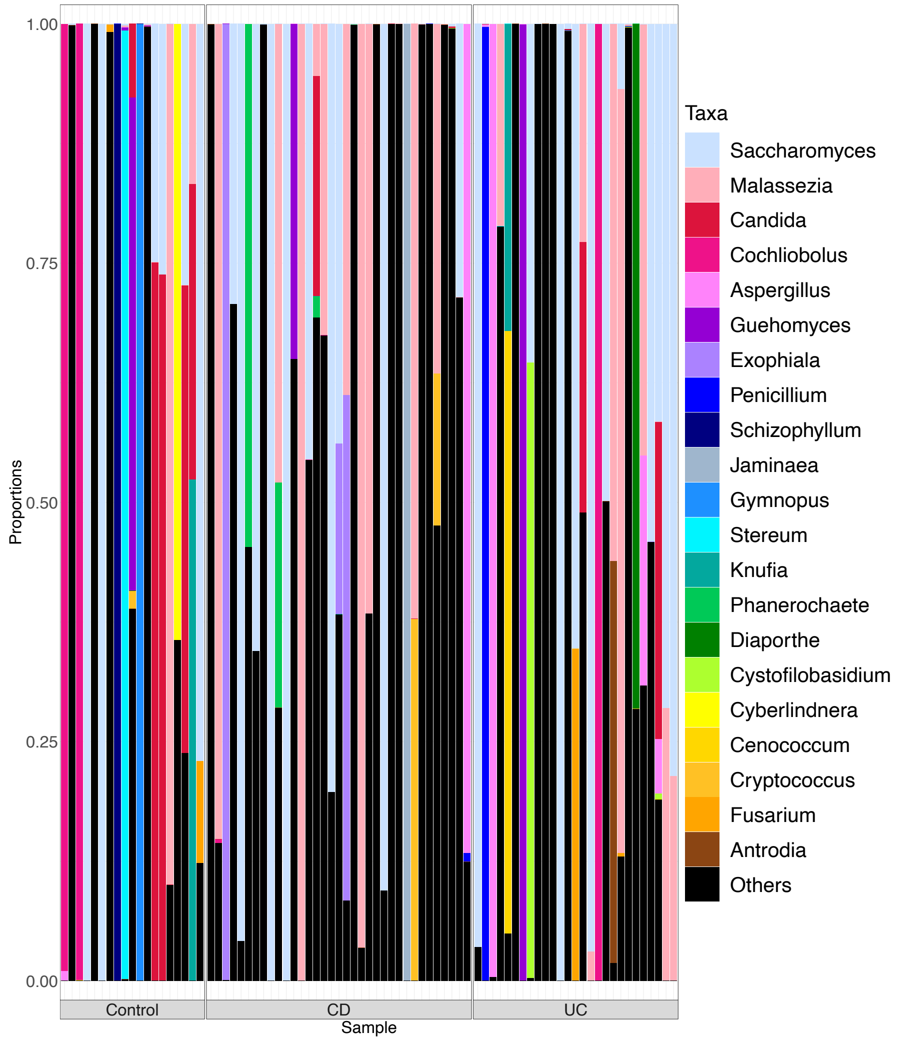

Figure S5 Taxa summary at genus level of biopsy samples (fungal 18S rRNA, British cohort). *Terminal ileum (n = 25), transverse colon (n = 28) and sigmoid colon (n = 28).*

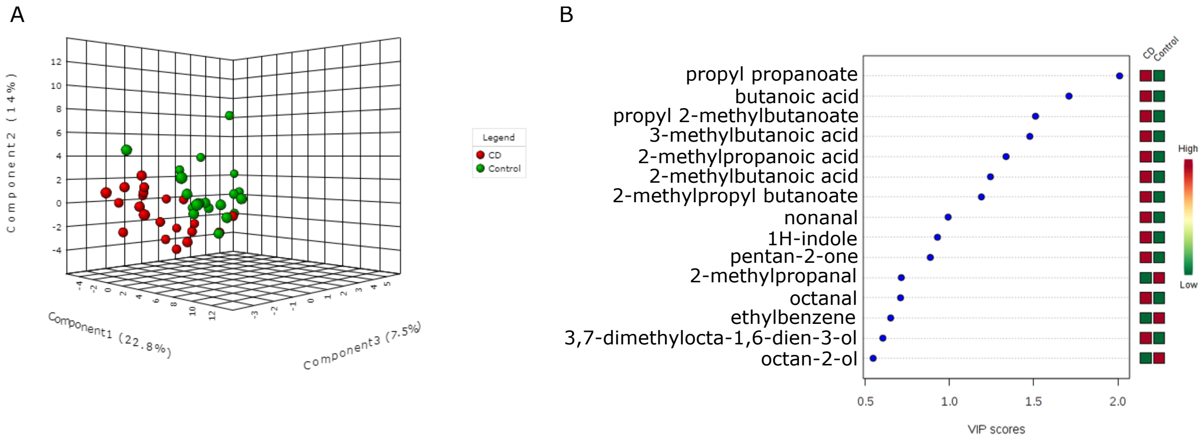

***Figure S6 Volatile organic compounds (VOCs) analysis results**. **A** PLS-DA (partial least squares projection to latent structures-discriminant analysis) plot comparing Crohn's disease (CD) vs Control showed a separation between these categories (p=0.04), p value was calculated with a permutation test. **B** Variables important in projection (VIP) of metabolites identified by PLD-SA that drove the separation when comparing CD vs control.*

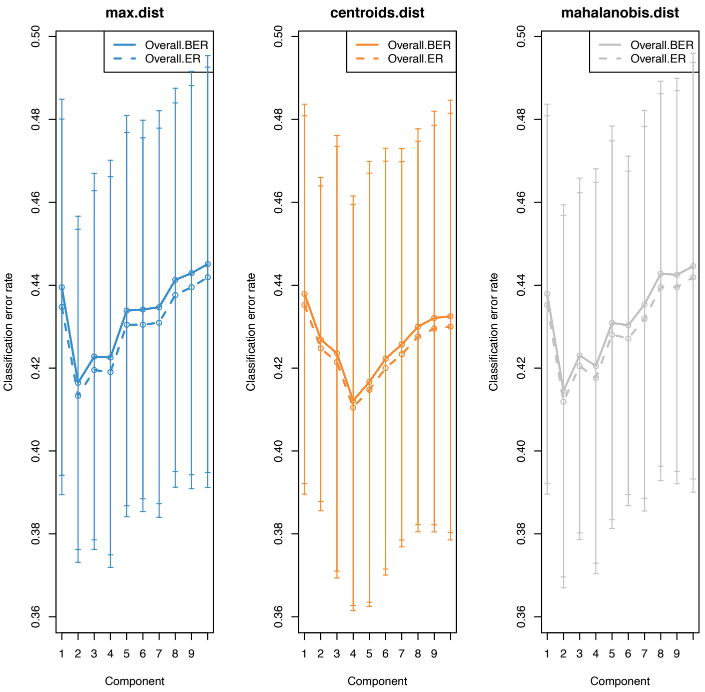

***Figure S7 Error rate (ER) and Balanced Error Rate (BER) results.** These refer to the model computed by Diablo (Mixomics) comparing Crohn's disease (CD) vs Controls (British Cohort) integrating fungal 18S rRNA, bacteria 16S rRNA and volatile organic compounds (VOCs) data from stool.*

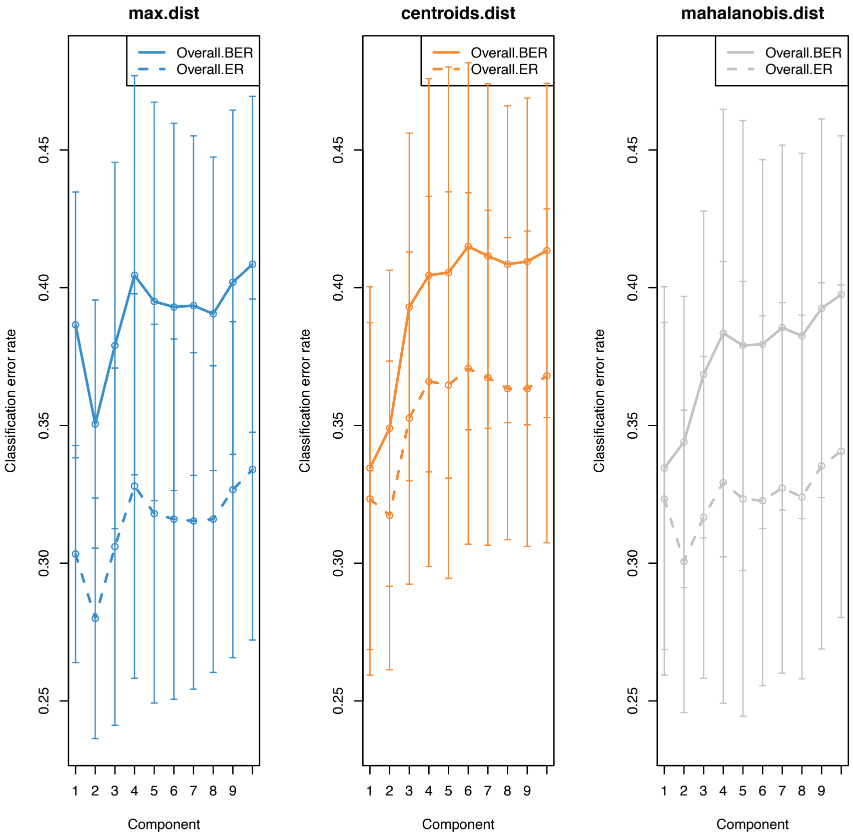

Figure S8 Error rate (ER) and Balanced Error Rate (BER) results. *These refer to the model computed by Diablo (Mixomics) comparing Crohn's disease (CD) active vs Controls (British Cohort) integrating fungal 18S rRNA, bacteria 16S rRNA and volatile organic compounds (VOCs) data from stool.*

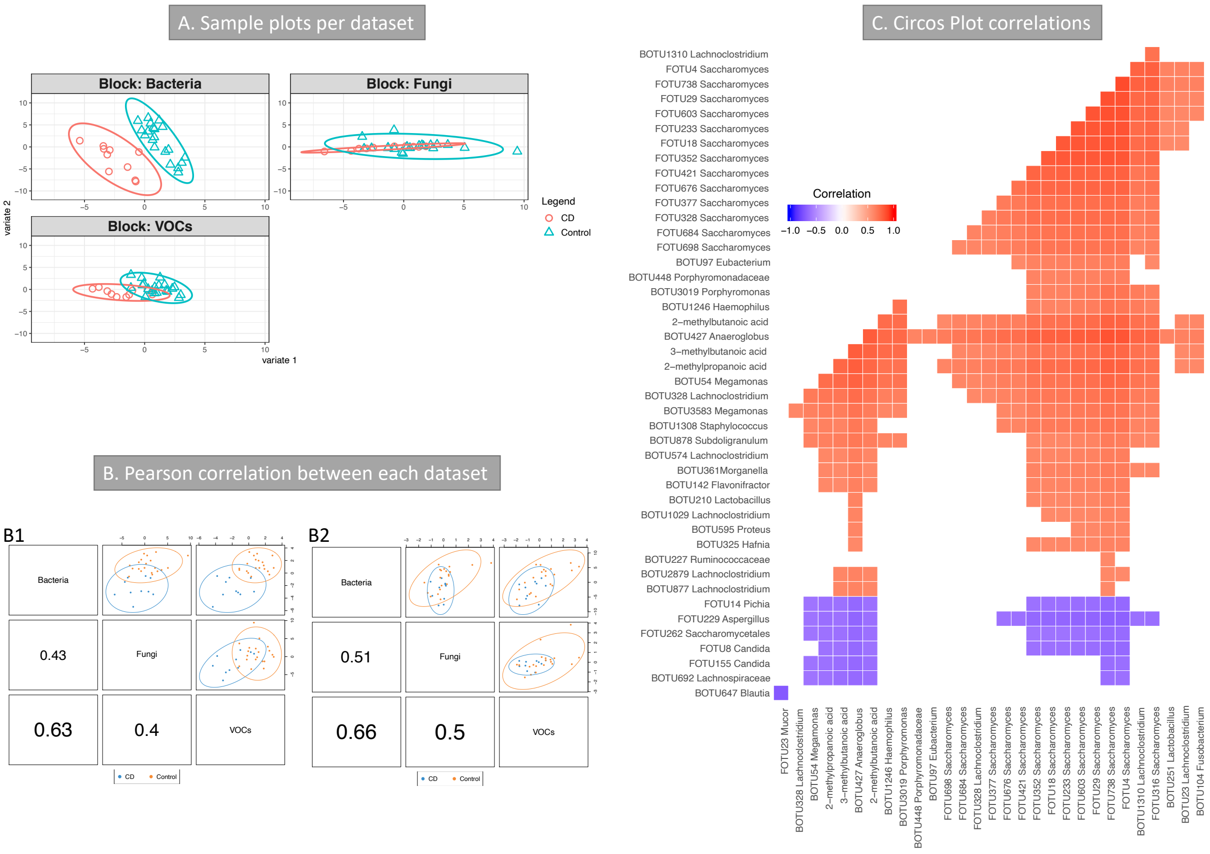

Figure S9 Integration of metabolomics (VOCs) and metagenomics (bacterial and fungi) stool data (Crohn's disease (CD) active vs Controls). *Data were from the British cohort.* ***A*** *three omics sample plots shown separately.* ***B*** *Pearson correlation of the three data set for each component.* ***C*** *Correlations (cut-off 0.6) produced in the circo plot are summarised in a heatmap (sorted by correlation values) in the right.*

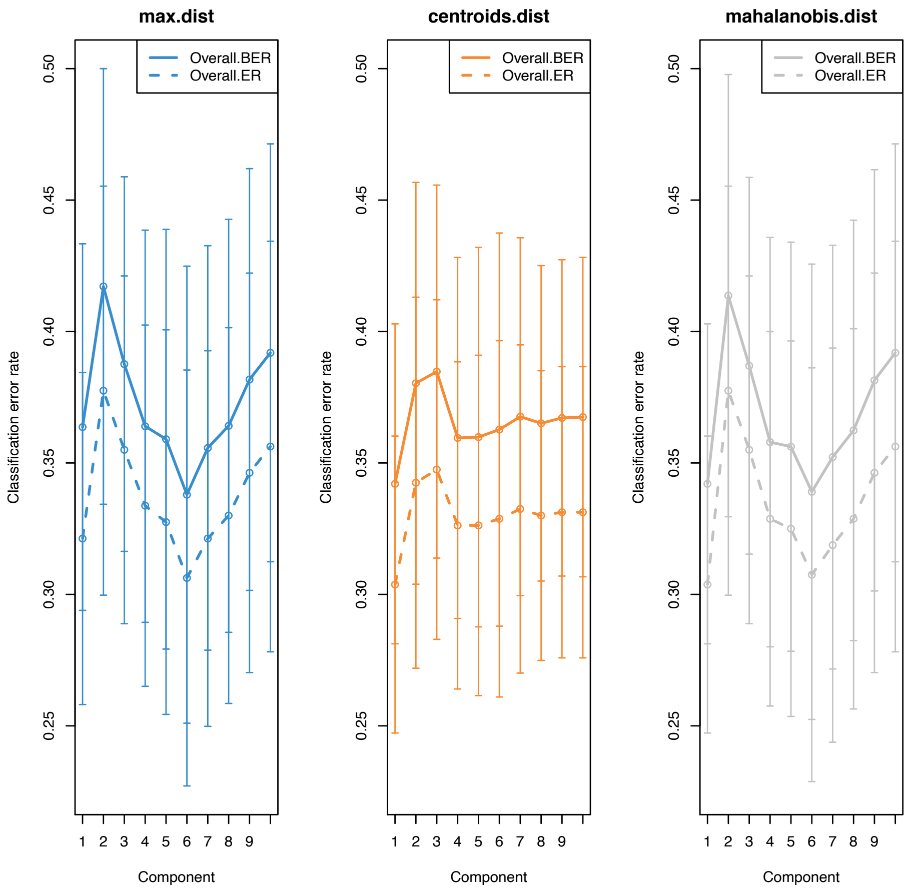

Figure S10 Error rate (ER) and Balanced Error Rate (BER) of the model computed by Diablo (Mixomics). *This was obtained comparing Crohn's disease (CD) vs Controls (British Cohort) integrating fungal 18S rRNA, bacteria 16S rRNA data from transverse colon mucosa and volatile organic compounds (VOCs) data from stool.*

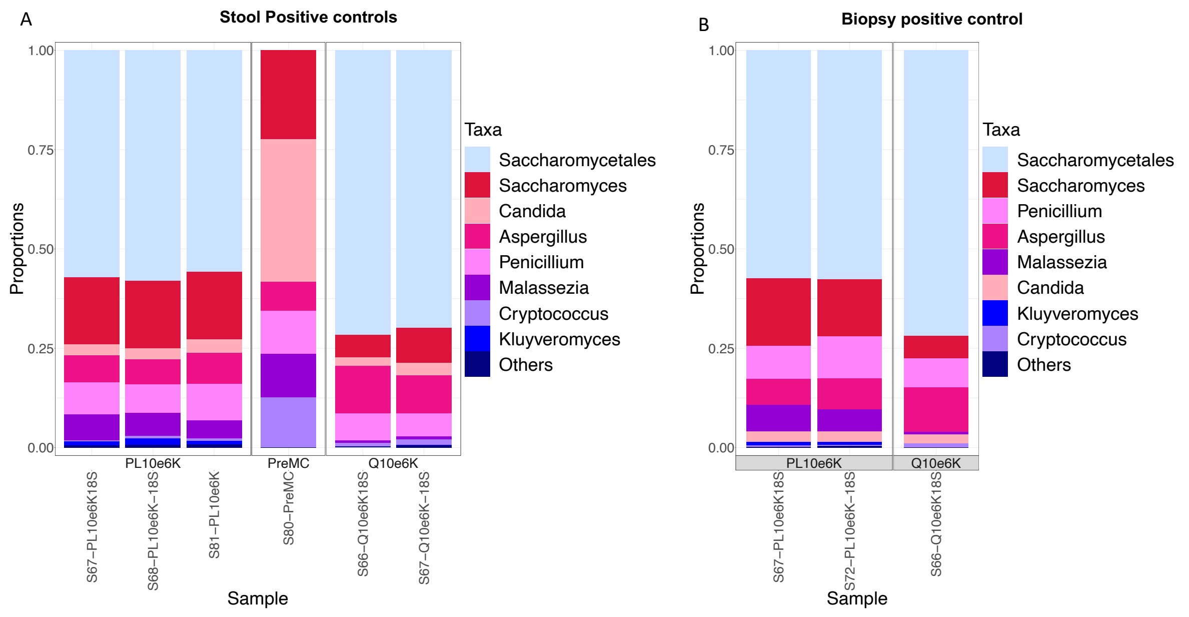

Figure S11 Mycobiome analysis positive controls results. *Taxa summary at genus level of positive controls for 18S rRNA. The positive controls are the same used in* (1). *PL10e6K and Q10e6K refers to spiked stool samples extracted with two different kits and PreMC refers to a Mock Community. Datasets were analysed according to site; however, the samples were run separately, with Liverpool stool in one run, Liverpool biopsies across 2 runs, one of these with Dutch healthy controls and Dutch Crohn's disease samples in a different run. Therefore, S66-Q10e6K18S and S67-PL10 e6K18S across the two bar charts A and B are the same samples.*

References:

1. Frau A, Kenny JG, Lenzi L, *et al.* DNA extraction and amplicon production strategies deeply inf luence the outcome of gut mycobiome studies. Sci. Rep. 2019;9:9328.
